# Optimal priming of poxvirus vector (NYVAC)-based HIV vaccine regimens for T cell responses requires three DNA injections. Results of the randomized multicentre EV03/ANRS VAC20 Phase I/II Trial

**DOI:** 10.1371/journal.ppat.1008522

**Published:** 2020-06-26

**Authors:** Yves Lévy, Christine Lacabaratz, Kim Ellefsen-Lavoie, Wolfgang Stöhr, Jean-Daniel Lelièvre, Pierre-Alexandre Bart, Odile Launay, Jonathan Weber, Bernd Salzberger, Aurélie Wiedemann, Mathieu Surenaud, David M. Koelle, Hans Wolf, Ralf Wagner, Véronique Rieux, David C. Montefiori, Nicole L. Yates, Georgia D. Tomaras, Raphael Gottardo, Bryan Mayer, Song Ding, Rodolphe Thiébaut, Sheena McCormack, Geneviève Chêne, Giuseppe Pantaleo

**Affiliations:** 1 Vaccine Research Institute, Université Paris-Est Créteil, Faculté de Médecine, INSERM U955, équipe 16, Créteil, France; 2 Assistance Publique-Hôpitaux de Paris, Groupe Henri-Mondor Albert-Chenevier, Service d’Immunologie Clinique, Créteil, France; 3 Centre Hospitalier Universitaire Vaudois (CHUV), Lausanne, Switzerland; 4 MRC Clinical Trials Unit at UCL, London, United Kingdom; 5 Université de Paris, Faculté de médecine Paris Descartes; Inserm, CIC 1417, F-CRIN I-REIVAC; Assistance Publique-Hôpitaux de Paris, CIC Cochin Pasteur, Paris, France; 6 Imperial College London, London, United Kingdom; 7 University Hospital, Institute of Clinical Microbiology and Hygiene, University of Regensburg, Regensburg, Germany; 8 Department of Medicine & Department of Global Health, University of Washington, Fred Hutchinson Cancer Research Center Seattle, Washington, United States of America; 9 ANRS, Paris, France; 10 Department of Surgery, Duke Human Vaccine Institute, Duke University Medical Center, Durham, North Carolina, United States of America; 11 Vaccine and Infectious Disease Division, Fred Hutchinson Cancer Research Center, Seattle, Washington, United States of America; 12 Public Health Sciences Division, Fred Hutchinson Cancer Research Center, Seattle, Washington, United States of America; 13 EuroVacc Foundation, Lausanne, Switzerland; 14 Inserm, Bordeaux Population Health Research Center, UMR 1219, University Bordeaux, ISPED, CIC 1401-EC, Univ Bordeaux, Bordeaux, France; 15 CHU de Bordeaux, pôle de santé publique, Bordeaux, France; 16 INRIA SISTM, Talence, France; 17 Swiss Vaccine Research Institute, Lausanne University Hospital, University of Lausanne, Lausanne, Switzerland; Emory University, UNITED STATES

## Abstract

DNA vectors have been widely used as a priming of poxvirus vaccine in prime/boost regimens. Whether the number of DNA impacts qualitatively or quantitatively the immune response is not fully explored. With the aim to reinforce T-cell responses by optimizing the prime-boost regimen, the multicentric EV03/ANRS VAC20 phase I/II trial, randomized 147 HIV-negative volunteers to either 3xDNA plus 1xNYVAC (weeks 0, 4, 8 plus 24; n = 74) or to 2xDNA plus 2xNYVAC (weeks 0, 4 plus 20, 24; n = 73) groups. T-cell responses (IFN-γ ELISPOT) to at least one peptide pool were higher in the 3xDNA than the 2xDNA groups (91% and 80% of vaccinees) (P = 0.049). In the 3xDNA arm, 26 (37%) recipients developed a broader T-cell response (Env plus at least to one of the Gag, Pol, Nef pools) than in the 2xDNA (15; 22%) arms (primary endpoint; P = 0.047) with a higher magnitude against Env (at week 26) (P<0.001). In both groups, vaccine regimens induced HIV-specific polyfunctional CD4 and CD8 T cells and the production of Th1, Th2 and Th17/IL-21 cytokines. Antibody responses were also elicited in up to 81% of vaccines. A higher percentage of IgG responders was noted in the 2xDNA arm compared to the 3xDNA arm, while the 3xDNA group tended to elicit a higher magnitude of IgG3 response against specific Env antigens. We show here that the modulation of the prime strategy, without modifying the route or the dose of administration, or the combination of vectors, may influence the quality of the responses.

## Author summary

Development of a safe and effective HIV-1 vaccine would undoubtedly be the best solution for the ultimate control of the worldwide AIDS pandemic. To date, only one large phase III trial (RV144 Thai study) showed a partial and modest protection against HIV infection. This result raised hope in the field and encouraged the development of vaccines or strategies in order to improve vaccine efficacy. Several vaccine strategies designed to elicit broad HIV-specific T cells and/or neutralizing antibodies to prevent HIV-1 transmission are under evaluation. Among diverse candidate vaccines, the safety and immunogenicity of multi-gene DNA-based and Pox-virus derived vaccines have been evaluated in several clinical studies. The present study was designed to optimize the combination of these two vaccines with the aim of determining the optimal number of DNA primes for a poxvirus-based HIV vaccine regimen. We show here that the prime boost combination is highly immunogenic and that the number of DNA primes induces differentially T cell and antibody responses. A better priming of poxvirus-based vaccine regimens for T cells is obtained with 3 DNA injections. Our results contribute and extend data of several preclinical studies pointing out the potential interest of DNA as a prime capable not only of improving immune responses but also of imprinting the long-term responses to boost vaccines.

## Introduction

Encouraging results from the RV 144 trial showed a modest but statistically significant, i.e. 31% reduction in the rate of HIV infection in vaccinated healthy volunteers receiving a prophylactic vaccine [1]. Nevertheless, to date, no effective therapeutic or prophylactic HIV vaccines are available.

Several vaccine strategies designed to elicit broad HIV-specific T cells and/or neutralizing antibodies [2, 3] to prevent HIV-1 transmission are under evaluation [4]. Among diverse candidate vaccines, the safety and immunogenicity of multi-gene DNA-based vaccines have been evaluated in several clinical phase I/II studies in the last years [5–12]. Different doses, intervals of administration and mode of delivering DNA vaccines have confirmed that DNA based vaccines in combination with live-vector in prime-boost regimens are safe and able to induce HIV-specific potent, polyfunctional and durable T-cell responses, as assessed by IFN-γ ELISPOT and cytokine production assays [5–12].

The best-studied vaccine vectors in humans are the poxviruses. Vaccinia virus engineered with HIV-1 genes has been shown to induce virus-specific cellular and humoral immune responses in immunized macaques and protection against simian immunodeficiency virus (SIV) infection when immunization with such constructs is followed with boosting by recombinant proteins [13–15]. Particular attention has been focused on poxviruses with limited *in vivo* replicative capacity and, therefore, limited pathogenicity, e.g. MVA or NYVAC, which have deletions of the genes associated with pathogenicity, or avian poxviruses, which do not complete an entire replication cycle in human cells but initiate protein synthesis and thus elicit immune responses. Clinical experience has been reported with a non-replicating poxvirus construct, which expresses several antigens derived from the malaria parasite *Plasmodium Falciparum* (NYVAC-Pf7) [16, 17] or expressing different HIV genes [9, 18, 19].

While several prime-boost combinations are under preclinical or clinical development, whether the number of DNA administered as a prime of recombinant vectors might impact or modulate the magnitude and the breadth of vaccine responses has not been fully evaluated. Limited data from a phase I DNA prime/Adenovirus 5 boost study performed within the Vaccine Research Center (VRC) program, showed that the cellular response was significantly broader (Env plus Gag) in volunteers that were primed by three DNA immunizations [5, 20, 21].

The present study was built on previous phase I studies performed within the EuroVacc vaccine program (EV01 and EV02 phase I studies) showing that two primes with a DNA-C vaccine improved vaccine responses to NYVAC-C boost in HIV-negative volunteers as compared to those who received NYVAC administration alone [9, 18, 19]. In order to assess the value of a third DNA vaccination in terms of eliciting a broader and more potent immune response, the randomized EV03/ANRS VAC20 trial aimed at comparing a regimen combining three primes with DNA-C (3xDNA) followed by one NYVAC-C boost (1xNYVAC) to two DNA-C primes (2xDNA) followed by two NYVAC-C boosts (2xNYVAC).

## Results

### Recruitment and follow-up

**Fig 1:**
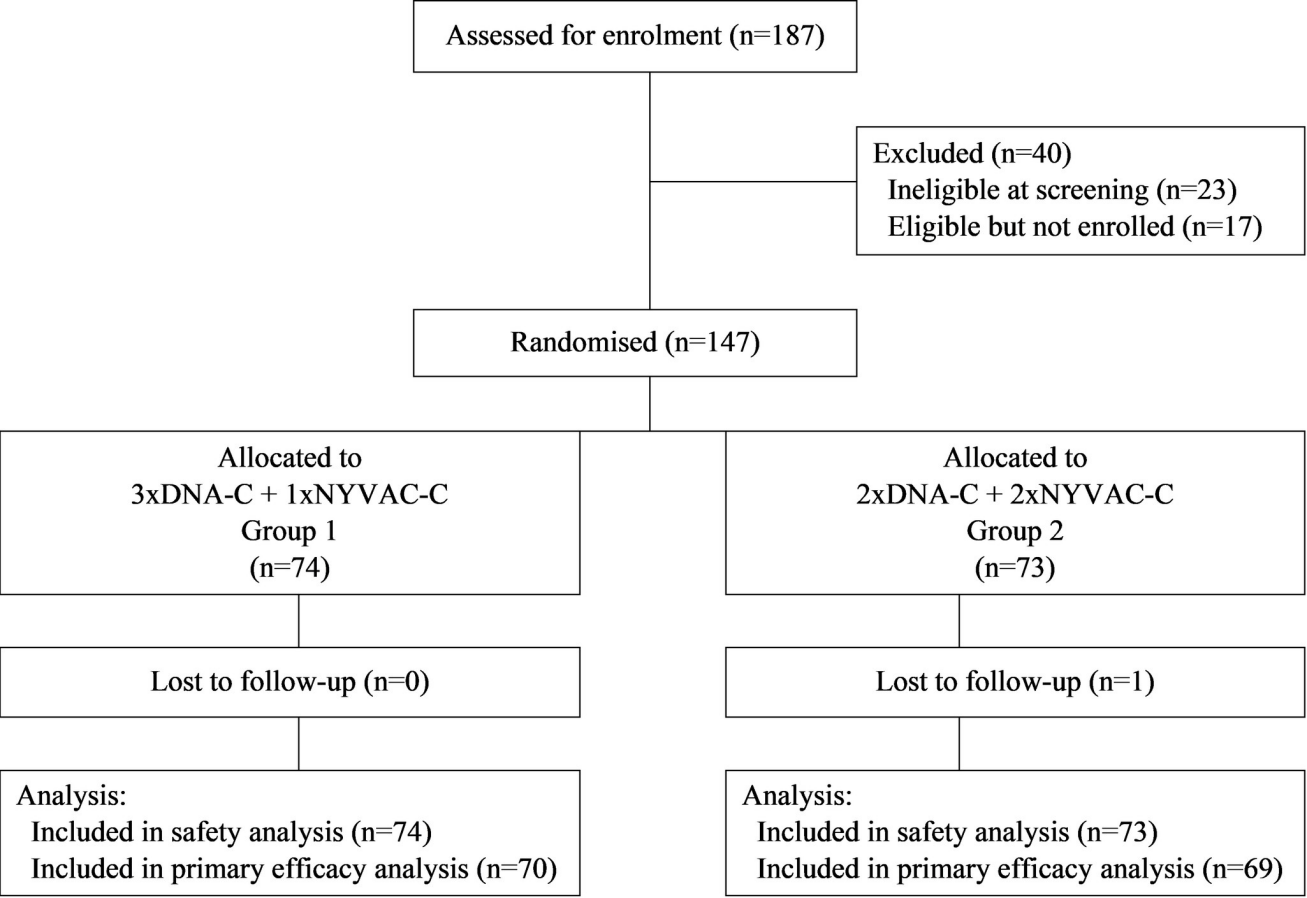
Consort flow diagram, EV03/ANRS VAC20 Phase I/II Trial.

Of the 187 volunteers screened, 147 participants were enrolled and randomly assigned to 2xDNA group (n = 73) or 3xDNA (n = 74) (Fig 1). Twenty-three were not eligible at screening for clinical or biological reasons and 17 were eligible but not enrolled (13 declined to participate and 4 because enrolment had closed). Demographics and clinical characteristics of participants are shown in Table 1 and did not differ between groups. Overall, 140/147 participants (95%) completed all 4 immunisations. One participant discontinued following the first DNA administration due to a related adverse event (AE; see below), one missed the second DNA injection due to unrelated AE but received the following immunizations, one participant missed the third DNA boost due to unavailability, but received the scheduled NYVAC boost. Two participants missed the fourth NYVAC immunisation: one was discontinued following a related adverse event (see below) and the second was not available. Two participants missed the third and fourth NYVAC immunizations for personal reasons, but attended follow-up.

**Table 1:** Demographic characteristics of participants, EV03/ANRS VAC20 Phase I/II Trial.

	3 DNA + 1 NYVAC		2 DNA + 2 NYVAC		Total	
Demographic characteristics	N = 74		N = 73		N = 147	
Geographical region, n (%)						
France	37	(50)	37	(51)	74	(50)
Switzerland, UK, Germany	37	(50)	36	(49)	73	(50)
Gender, n (%)						
Male	38	(51)	38	(52)	76	(52)
Female	36	(49)	35	(48)	71	(48)
Mean age in years (SD)	39	(11)	37	(10)	38	(11)
Median age in years (IQR)	39	(26; 49)	37	(29; 46)	38	(28; 47)
Range of age in years [min-max]	[19; 55]		[18; 55]		[18; 55]	
Age in categories, n (%)						
<25	11	(15)	11	(15)	22	(15)
[25;35]	17	(23)	22	(30)	39	(27)
[35;45]	20	(27)	18	(25)	38	(26)
≥ 45	26	(35)	22	(30)	48	(33)
Ethnic, n (%)						
white	74	(100)	69	(95)	143	(97)
black	0		1	(1)	1	(1)
asian/pacific	0		1	(1)	1	(1)
other	0		2	(3)	2	(1)
Tobacco consumption, n (%)						
no	43	(58)	35	(48)	78	(53)
yes	13	(18)	18	(25)	31	(21)
yes but stopped	18	(24)	20	(27)	38	(26)
Alcohol consumption, n (%)						
no	26	(35)	19	(26)	45	(31)
yes	48	(65)	54	(74)	102	(69)

### Safety

**Fig 2:**
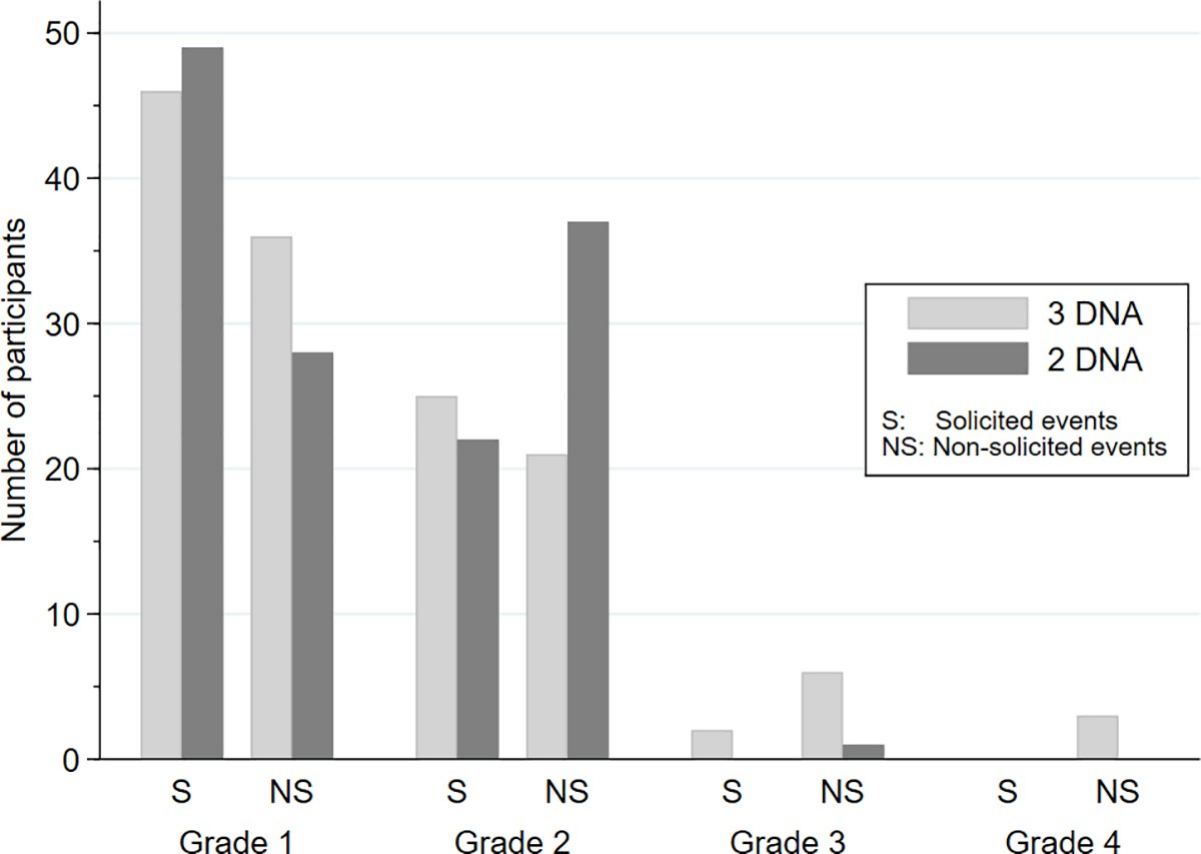
Frequencies of participants with solicited and non-solicited events per vaccine groups, EV03/ANRS VAC20 Phase I/II Trial. The maximum grade is shown per participant and event category. Differences between groups: p = 0.32 and p = 0.56 for solicited and non-solicited events, respectively (rank tests).

All participants enrolled received at least one vaccine dose and were included in the safety evaluations (n = 147). Globally, tolerance of vaccines was good. The frequency and severity of solicited adverse events were not significantly different between groups (Fig 2 & S1 Table). There were minor abnormalities in hematologic and hepatic profiles but no differences between the 2 groups. Two participants among 147 (both in the 3xDNA group) experienced an adverse event attributable to immunisation that led to discontinuation of the schedule (1 loss of consciousness after the first DNA, and 1 severe vaso-vagal reaction after the third DNA) (difference between groups: p = 0.50). All other grade 3 or 4 non-solicited adverse events were regarded as unrelated to the vaccines (Fig 2).

### T-cell Immunogenicity of vaccine strategies

Of the 147 randomized participants, 7 had missing primary endpoint data: 2 in the 3xDNA group had no samples available, and 5 (2 and 3, in 3xDNA and 2xDNA groups, respectively) had invalid results (negative control ≥ 50 SFU/10^6^ cells). Another participant (2xDNA) was excluded because of baseline responses to the peptide pools Env1, Env2, Gag1, Pol1, and Pol2. Of the remaining 139 individuals, 134 completed all scheduled immunizations (per protocol), 67 in the 3xDNA group, and 67 in the 2xDNA group.

Overall, the proportion of responders to any HIV peptide pools at weeks 26/28 was 86% (119/139 participants). The proportions were 91% (64/70) and 80% (55/69) in the 3xDNA and 2xDNA groups, respectively (chi-squared test: P = 0.049; risk difference: 11.7%; 95%CI: 0.2–23.3%). In the Per Protocol analysis, the proportions were 94% (63/67) and 81% (54/67), respectively (P = 0.020; risk difference: 13.4%; 95%CI: 2.4–24.5%). The number (%) of participants in the 3 categories: no response, Env only, and Gag/Pol/Nef (±Env) were 6 (9%), 37 (53%), and 27 (39%) in 3xDNA, and 14 (20%), 39 (57%), and 16 (23%) in 2xDNA (n = 139, overall difference p = 0.046).

At week 26, the proportion of responders to Env was 87% and 72% (P = 0.034) in the 3xDNA and 2xDNA groups, respectively (Table 2). At the same time point, responders to either Gag, Pol or Nef pools were 31% and 18%, respectively (P = 0.07). The proportions of responders to individual pools are reported in S2 Table.

**Table 2:** Proportions of responders to any peptide pool (Env and/or Gag/Pol or Nef; Overall responses), to Env pools (Env) or Gag/Pol/Nef (Gag/Pol/Nef) at weeks (26/28; primary time points) and week 48 and 72, EV03/ANRS VAC20 Phase I/II Trial.

Pools	Week 26		Week 28		Week 48		Week 72	
	3 DNA	2 DNA	3 DNA	2 DNA	3 DNA	2 DNA	3 DNA	2 DNA
**Overall**	58/67	49/67	61/69	54/67	55/66	47/70	48/67	38/70
	(87%)	(73%)	(88%)	(81%)	(83%)	(67%)	(72%)	(54%)
	P = 0.053	P = 0.21		P = 0.029		P = 0.036	
**Env**	58/67	48/67	59/69	54/67	54/66	43/70	47/67	34/70
	(87%)	(72%)	(86%)	(81%)	(82%)	(61%)	(70%)	(49%)
	P = 0.034	P = 0.45		P = 0.009		P = 0.01	
**Gag, Pol or Nef**	21/67	12/67	21/69	13/67	18/66	18/70	16/67	13/70
	(31%)	(18%)	(30%)	(19%)	(27%)	(26%)	(24%)	(19%)
	P = 0.07	P = 0.14		P = 0.84		P = 0.45	

The primary immunogenicity endpoint (frequency of responders to Env and at least one of the Gag/Pol/Nef pools) was measured at week 26 and 28. Overall, 26/70 (37%) and 15/69 (22%) participants from the 3xDNA and 2xDNA groups, respectively, reached the primary endpoint (chi-squared test: P = 0.047; risk difference: 15.4% (95% CI 0.5–30.3%); risk ratio: 1.7 (95% CI 1.0–2.9)). In the Per Protocol analysis, these proportions were 25/67 (37%) and 15/67 (22%), respectively (chi-squared test: P = 0.059; risk difference: 14.9% (95% CI -0.4–30.2%); risk ratio: 1.7 (95% CI 1.0–2.9)).

**Fig 3:**
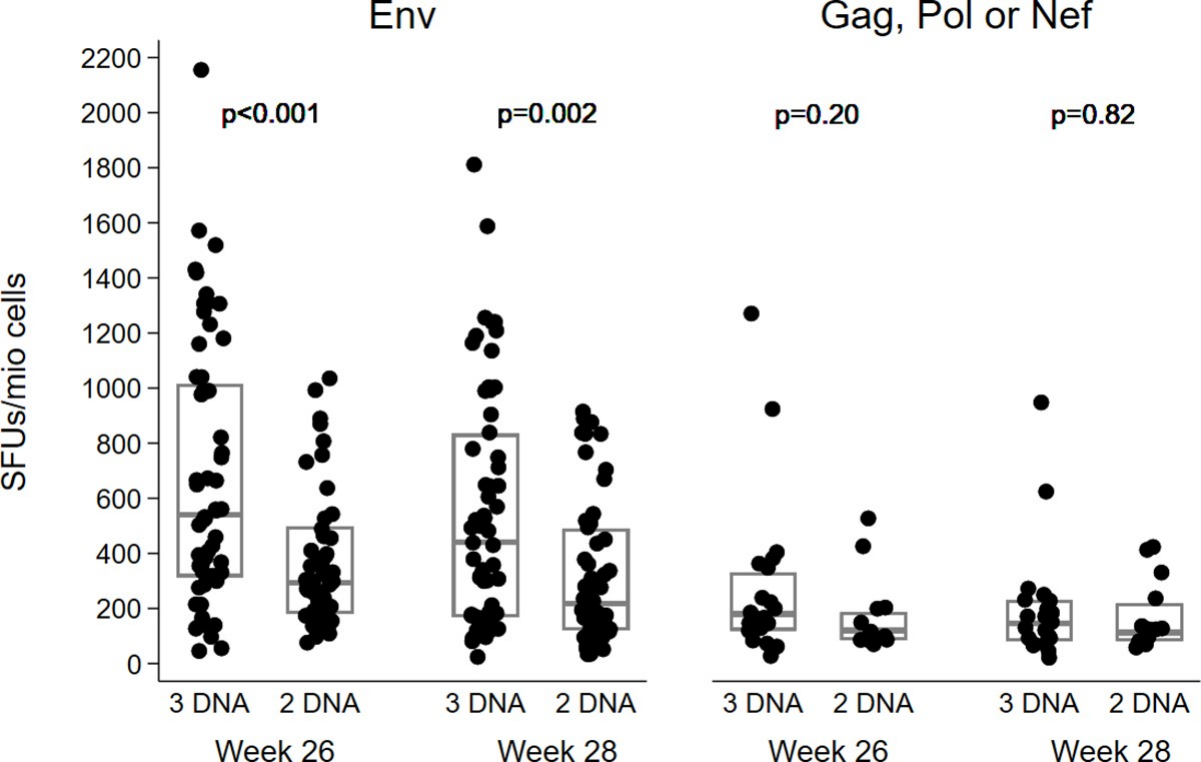
Magnitude of T-cell responses in vaccine groups at the primary end points (weeks 26/28) measured by IFN-γ ELISPOT assay, EV03/ANRS VAC20 Phase I/II Trial. Individual magnitude (SFC/10^6^ PBMC) of vaccine-induced T cell responses against Env (left panel) or Gag, Pol, and Nef (right panel) in the two study groups at weeks 26 and 28. Boxes represent median values with IQR. Comparisons between groups were made using rank tests.

At week 26, the median number (IQR) of SFU/10^6^ cells against Env pools was 539 (315–1013) in the 3xDNA group which was significantly higher than in the 2xDNA group (294; 182–496) (ITT analysis, P<0.001). At the same time point, responses to Gag, Pol or Nef pools were 180 (120–331) and 120 (86–187), respectively (P = 0.20). The overall magnitude of responses had a median of 545 (340–1101) versus 336 (185–488) SFUs/10^6^ cells in the 3xDNA and 2xDNA arms, respectively (P<0.001). At week 28, the differences between the groups remained significant and response magnitudes were 442 (170–833) and 217 (123–488) SFU/10^6^ cells against Env (P = 0.003) (Fig 3), and 445 (170–855) and 235 (123–505) SFU/10^6^ cells overall (p = 0.006).

**Fig 4:**
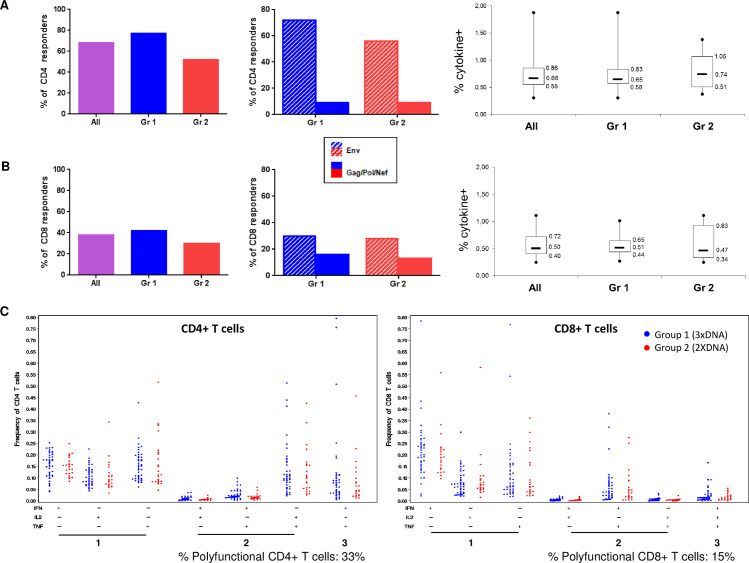
Frequency and magnitude of CD4^+^ and CD8^+^ T-cell responses in vaccine groups at the primary end points (weeks 26/28) measured by ICS assay, EV03/ANRS VAC20 Phase I/II Trial. A. Left panel: Frequency of positive response for CD4+ T cells producing at least one cytokine (IFN-γ, TNF-α or IL2) to at least one HIV pool in all participants (n = 72) or in each group (n = 43 in 3xDNA group, blue; n = 23 in 2xDNA group, red). Middle panel: Frequency of positive response for CD4+ T cells producing at least one cytokine to Env (hatched histograms) and Gag/Pol/Nef (solid histograms) in each group. Right panel: Magnitude of CD4+ T cell response in all participants and in each group. Boxes represent median values with IQR and whiskers represent min and max values. B. Left panel: Frequency of positive response for CD8+ T cells producing at least one cytokine (IFN-γ, TNF-α or IL2) to at least one HIV pool in all participants (n = 72) or in each group (n = 43 in 3xDNA group, blue; n = 23 in 2xDNA group, red). Middle panel: Frequency of positive response for CD8+ T cells producing at least one cytokine to Env (hatched histograms) and Gag/Pol/Nef (solid histograms) in each group. Right panel: Magnitude of CD8+ T cell response in all participants and in each group. Boxes represent median values with IQR and whiskers represent min and max values. C. Polyfunctional profile of CD4+ (left panel) and CD8+ (right panel) T cell populations in each group (3xDNA in blue, 2xDNA in red). All the possible combinations of the responses are shown on the x axis, whereas the percentage of the functionally T cell populations are shown on the y axis.

ICS were performed at W26/28 on 72 participants (n = 43 3xDNA group, n = 23 2xDNA group) with a positive IFN-γ ELISPOT response to Env1/Env2 pools showing a magnitude of response ≥ 200 SFU/10^6^ cells. Globally, the frequency of CD4^+^ T cells producing at least one cytokine to at least one HIV pool was detected in 68% of vaccinees. The majority of CD4^+^ T cell responses was directed against Env (72% and 56% in 3xDNA and 2xDNA groups, respectively) while only 9% were directed to Gag/Pol/Nef. The magnitude of CD4^+^ T cell responses (median [IQR]) was 0.66% [0.55–0.86] without difference between groups (Fig 4A).

For CD8^+^ T cell responses, 38% of vaccinees were responders to at least one HIV pool and the magnitude (median [IQR]) was 0.50% [0.40–0.72] without difference between groups. In each group, 28–30% of vaccinees responded to Env and 13–16% to Gag/Pol/Nef (Fig 4B).

The analysis of polyfunctionality showed that, among HIV-specific CD4^+^ and CD8^+^ T cells, 33% and 15% produced at least 2 cytokines, respectively (Fig 4C).

**Fig 5:**
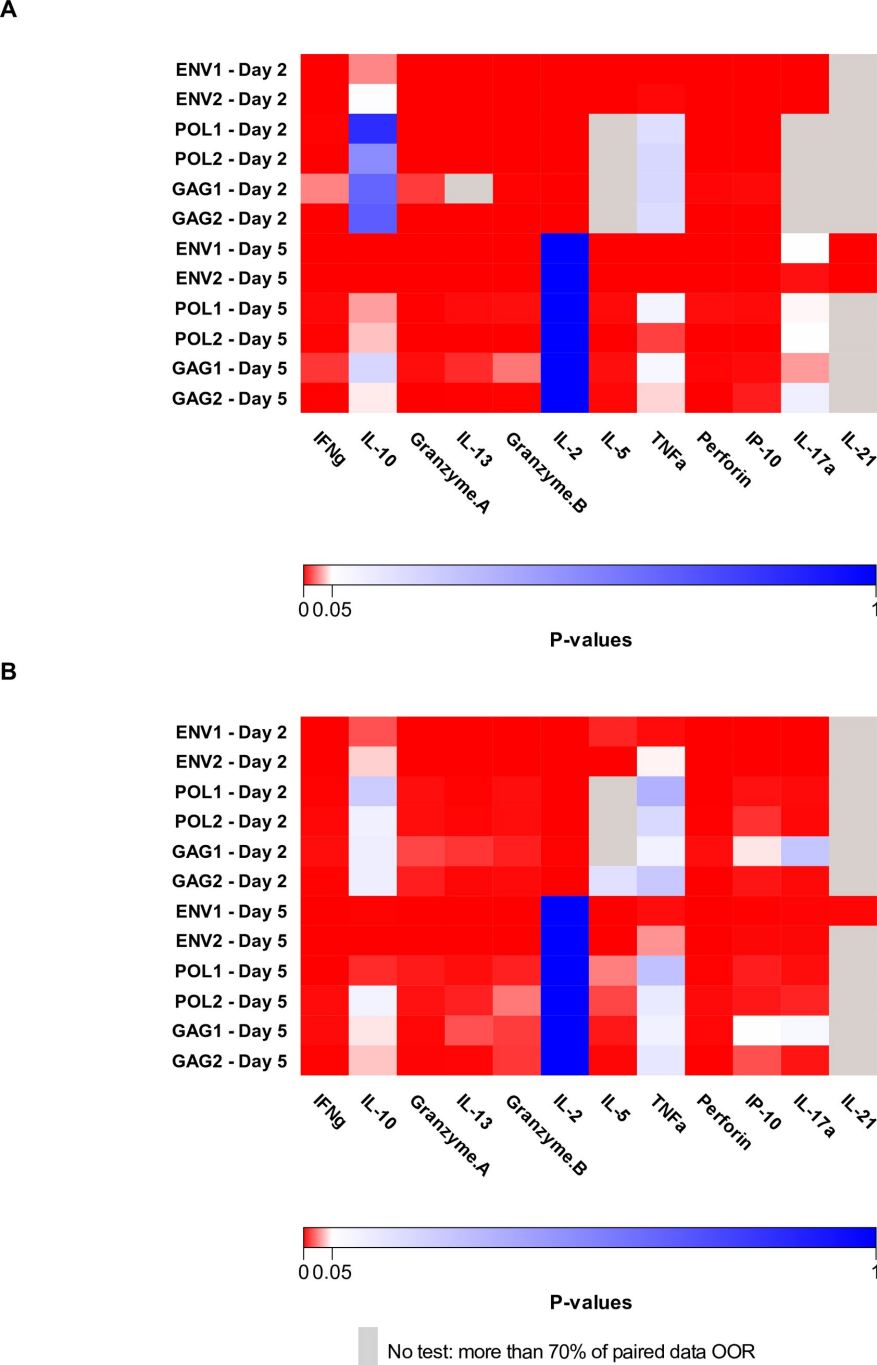
Multiplex cytokine measurements, EV03/ANRS VAC20 Phase I/II Trial. Heatmap of p values for W0-W26/28 comparisons of cytokine secretion after PBMC stimulation with each HIV peptide pool (Env1, Env2, Pol1, Pol2, Gag1 and Gag2) for 2 and 5 days from 49 participants (A. n = 29 3xDNA group, B. n = 20 2xDNA group). No comparison was performed when more than 70% of paired data were below the lower limit of quantification (grey squares).

We investigated the cytokine profile of vaccine-elicited cellular responses more broadly using a cytokine multiplex analysis in 49 participants who had completed the vaccine schedule (n = 29 3xDNA group, n = 20 2xDNA group). Fig 5 illustrates p values for W0-W26/28 comparisons. For both groups of participants, a significantly higher production of Th1 (IFN-γ, IL-2, TNF-α, IP-10), Th2 (IL-5, IL-10, IL-13), and Th17 (IL-17A, IL-21) cytokines as well as cytotoxic markers (GrzA, GrzB, Perforin) was found at W26/28 after stimulation of PBMC with at least one of the HIV pools.

### Neutralizing antibody responses

HIV neutralizing activity of antibody responses against Env was tested at week 26 in 120 participants. Overall, 14 (12%) individuals developed responses that neutralized one or both of the highly sensitive tier 1A viruses MN.3 and MW965.26. The proportion of responders between groups was not different and was 8/56 (14%) and 6/64 (9%) in the 3xDNA and 2xDNA groups, respectively (chi-squared test: P = 0.40). The magnitude of the responses was low in most responders (titre <100) although some participants had strong responses (n = 3 with titre >300) against one or more tier 1A viruses. No neutralization of tier 2 viruses was detected.

### Binding antibody responses

**Fig 6:**
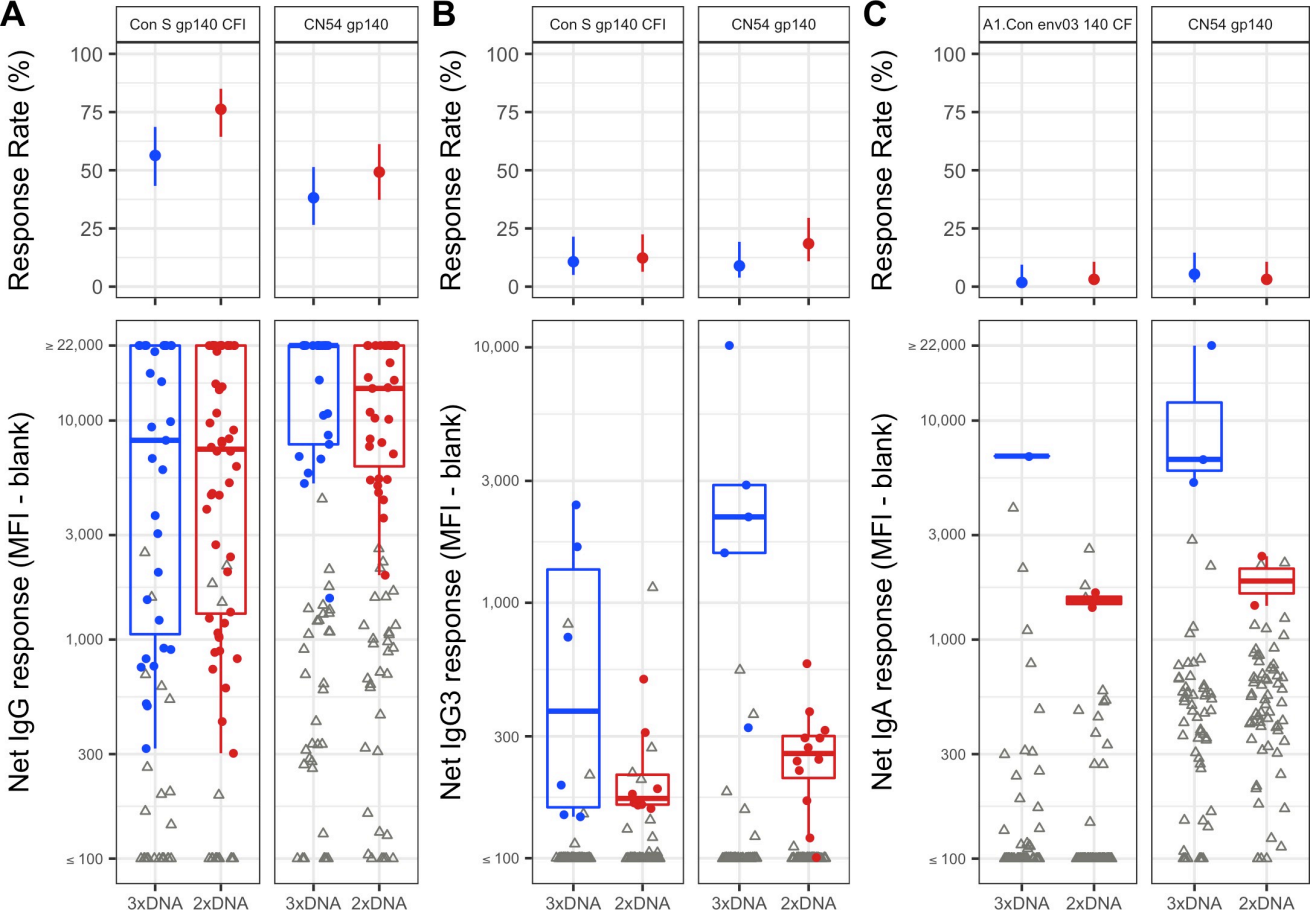
Binding Antibody Responses, EV03/ANRS VAC20 Phase I/II Trial. Response rates with 95% confidence intervals (top) and magnitude of response (bottom) at week 26 with open triangles for non-responders and closed circles for responders. A) IgG for Con S gp140 CFI and CN54 gp140, B) IgG3 for Con S gp140 CFI and CN54 gp140, C) IgA for A1.Con env03 140 CF and CN54 gp140.

HIV-1 binding antibody responses against consensus and CN54 vaccine strain gp140 antigens for IgG, IgG3, and IgA isotypes were tested at week 26. Overall there were moderate to high responses for IgG in both vaccine groups (38%—81% response rates) (Fig 6A, S1 Fig). For all antigens, there were higher response rates in the 2xDNA group, with significant differences for B.con.env03 140 CF (p = 0.0105), C.con.env03 140 CF (p = 0.0217), and Con S gp140 CFI (p = 0.0248). The 3xDNA group tended to elicit a higher magnitude of response in all but one antigen (C.Con.env03 140 CF), however none of the differences reached statistical significance (Fig 6B, S1 Fig). Overall there were low response rates for IgG3 (3%—18%) and there was little difference in response rates between the groups (Fig 6A). When considering the magnitude of the Env IgG3 response, the 3xDNA group elicited higher magnitude of response in all antigens, with a significant difference for antigen CN54 gp140 (p = 0.0013) (Fig 6B). There was little to no induction of Env specific IgA (Response rates of 0%—5%) (Fig 6A). At week 26 there was no significant correlations between IgA and IgG responses, either pooled overall (-0.08–0.16), or within group (3xDNA group (-0.15–0.05)), (2xDNA group (0.0–0.31)), (S2 Fig).

### Durability of the responses

Analyses of T-cell IFN-γ ELISPOT responses at week 48 and 72 showed that the proportion of responders against any HIV peptide pools either Env or Gag, Pol and Nef remained higher in the 3xDNA group and was 55/66 (83%) and 48/67 (72%) compared to 47/70 (67%) (P = 0.029) and 38/70 (54%) in the 2xDNA group (P = 0.036) (Table 2). These responses were mainly oriented against Env and were 82% and 70% in the 3xDNA group at week 48 and 72 as compared to 61% and 49% in the 2xDNA group at the same time points (P = 0.009 and P = 0.01 for comparisons between groups at weeks 48 and 72). At the same time points, the frequencies of responders against Gag/Pol or Nef pools were 27% and 24% in the 3xDNA group and 26% and 19% in the 2xDNA group (P = 0.84 and 0.45, respectively) (Table 2). The proportions of responders to individual pools are reported in S2 Table. Analysis of the magnitude of responses against either Env or Gag/Pol/Nef pools did not show differences between groups at week 48 and 72.

Therefore, although declining over time, the proportion of responders against any HIV peptide pools, remained significantly higher in the 3xDNA group as compared to the 2xDNA group throughout the study.

### Factors associated with vaccine-elicited immune responses

In the logistic regression analysis, 3xDNA arm was associated with a higher probability to reach the primary endpoint (i.e. responses to Env plus Gag/Pol or Nef) (Odds Ratio: 2.3; 95%CI:1.0–5.0; P = 0.042) (Table 3). Among other characteristics tested, older volunteers were less likely to be responders than younger ones (Odds Ratio: 0.5 per 10 years older; 95%CI: 0.3–0.9; P = 0.01). For neither alcohol nor smoking, as well as Bone Mass Index, there was a significant association with vaccine responses, neither in univariable or multivariable analyses. Interestingly, the presence of vaccinia antibodies at baseline was not associated with a poorer response to vaccination. The same characteristics were independently associated with the magnitude of responses assessed by ELISPOT analysis (Table 4).

**Table 3:** Multivariable logistic regression analysis of predictive factors of vaccine responses to Env and Gag/Pol/Nef, EV03/ANRS VAC20 Phase I/II Trial.

	Univariable models			Multivariable model		
Factor	Odds Ratio	95%CI	p	Odds Ratio	95%CI	p
3 DNA vs 2 DNA	2.1	1.0–4.5	0.048	2.3	1.0–5.0	0.042
Female vs male	0.8	0.4–1.7	0.62	0.7	0.3–1.6	0.38
Age (per 10 years older) ^¶^	0.6	0.4–0.9	0.011	0.5	0.3–0.9	0.010
Geographical region (France vs other)	0.6	0.3–1.3	0.20	1.1	0.4–2.8	0.84
Vaccinia antibodies*						
Binding Abs	0.9	0.4–1.9	0.78	1.3	0.5–3.0	0.60
Neutralizing Abs	1.4	0.6–3.2	0.44	2.1	0.8–5.5	0.14

Note

^¶^ linear association

* Results shown are for antibodies present versus not present; there also was no influence of antibody titre.

**Table 4:** Multivariate analysis of predictive factors of the magnitude of vaccine-elicited T cell responses, EV03/ANRS VAC20 Phase I/II Trial.

	Week 26			Week 28		
Factor	Coef^§^	95%CI	p	Coef^§^	95%CI	p
3 DNA vs 2 DNA	90	40–158	<0.001	63	17–126	0.004
Female vs male	-3	-29–33	0.85	-2	-30–39	0.93
Age (per 10 years older) ^¶^	-19	-32 - -4	0.016	-23	-37 - -7	0.006
Geographical region France vs other	8	-25–55	0.69	5	-30–57	0.81
Vaccinia antibodies *						
Binding Abs	-7	-34–33	0.71	27	-13–85	0.21
Neutralizing Abs	-2	-33–43	0.91	17	-23–76	0.46

Note

^§^ Coefficient: % difference in SFU/10^6^ cells after back-transformation

^¶^ linear association

* Results shown are for antibodies present versus not present; there also was no influence of antibody titre.

## Discussion

We report here results from a large phase I/II prophylactic HIV vaccine study comparing two prime-boost strategies in healthy volunteers with the aim of determining the optimal number of DNA primes for a poxvirus-based HIV vaccine regimen. Our results demonstrate that 3 DNA injections elicits a higher proportion of responders than 2 DNA primes for T cell responses. Globally, we confirm and expand substantially previous data by showing that the prime-boost combination of DNA-C and NYVAC-C elicited a high rate of T-cell responses to Env and/or Gag/Pol/Nef in up to 90% of volunteers at the primary time point (week 26/28). Furthermore, in comparison to two DNA-C primes, the addition of a third DNA improved the magnitude of T-cell responses to Env and expanded significantly the breadth of these responses to Gag/Pol/Nef epitopes. The durability of vaccine responses is also key for ensuring long-term protection. Although declining, the proportion of responders against any HIV antigens (Env ± Gag/Pol/Nef) remained significantly higher throughout the follow up and for 12 months following the last boost when the prime was optimized. We show that older participants were less likely to have T-cell responses (and lower response magnitude), whereas the presence of vaccinia antibodies was not associated with the type of response.

Several studies have assessed different doses, mode of delivery (IM, ID with or without electroporation) and timing of DNA vaccine [7–12, 18, 22–24]. Globally, DNA administration was shown to be safe and an excellent prime for Pox-based recombinant vectors administered with or without protein vaccines [7–12, 18]. Our study was built on previous phase I data testing different combinations of DNA-C and NYVAC-C vaccines in healthy volunteers. The preceding trials (EuroVacc EV01 and EV02) provided safety data on the highest practicable dose of the DNA-C and immunogenicity data. The EV02 trial compared a prime-boost regimen consisting on 2xDNA-C followed by 2xNYVAC-C compared to 2x NYVAC-C alone [9, 18]. Results showed that a higher proportion of volunteers (90%) that completed the prime-boost regimen had IFN-γ ELISPOT responses as compared to those that received only NYVAC-C (40%). Although these responses were robust and polyfunctional, they were highly oriented towards Env epitopes [9]. Therefore, in the present study, the primary immunogenicity endpoint was defined strictly to evaluate the breadth of the responses (frequency of responders to Env and at least one of the Gag/Pol/Nef pools) measured after the last boost of NYVAC-C (week 26 and 28). We show that a significantly higher proportion of volunteers have reached the primary endpoint in the 3xDNA-C prime (chi-squared test: P = 0.047; risk difference: 15.4% (95% CI 0.5–30.3%); risk ratio: 1.7 (95% CI 1.0–2.9)). Although the difference in the proportion of responders was lower than the 30% improvement considered clinically relevant, 3 DNA significantly improved the magnitude of T cell responses as assessed by IFN-γ ELISPOT assay. The analysis of cytokine production by ICS detected CD4 and CD8^+^ T cell responses in 68% and 38% of volunteers selected as responders to Env by ELISPOT.

The results reported here complement previous data reported recently by our group in Non-Human Primates (NHP) testing DNA-C as a prime of various boost strategies, i.e. NYVAC-C (tested in our clinical trial) or a replicating derivative-form of this vector (NYVAC-KC). In these studies, Pox vectors were administered either alone or combined with different HIV Env protein boosts given sequentially or simultaneously [25, 26]. Results clearly showed that the DNA-C priming was essential for shaping the breadth and the magnitude of B and T cell responses of the different boost strategies. This observation was reinforced by results from a parallel study [27] evaluating the immunogenicity of the two Pox vectors (NYVAC-C and its replicative derivative form NYVAC-KC) administered with a protein TV1-gp120 but without DNA-C priming. In these latter conditions, NYVAC-KC appeared more immunogenic than the non-replicative NYVAC-C, whereas no immunological differences were noted when these regimens were preceded by the DNA-C prime [25]. Globally, these preclinical data and results of our clinical study emphasize the importance of the DNA-C priming and its impact in the quality of the immune responses. In contrast to NHP studies, our study was designed to compare the two strategies (i.e: 3 or 2 DNA-C primes) on T cell responses at W26/28 and did not allow to analyse the role of the NYVAC-C boost in the increase of the magnitude of T-cell responses.

In depth analysis of correlates of HIV-1 risk in the large prophylactic RV144 Thai study revealed that a complex combination of effector T and B cell responses is associated with decreased HIV-1 risk [1, 28] (reviewed in [29]). Although there is a large consensus and focus on the value of broadly neutralizing antibodies in vaccine trials, there has been little demonstration that complex engineered Env antigens are capable of eliciting antibodies that neutralize tier 2 isolates in NHP trials until recently [30, 31]. Results from the Thai trial pointed out the need to harness the magnitude and functionality of binding antibody responses elicited by the vaccines and to extend the breadth and magnitude of T cell responses. Around 15% of volunteers from our study developed neutralizing antibodies against tier 1A viruses, the easier isolates to neutralize. This modest result is not unexpected and reinforces a large series of preclinical and clinical data suggesting the need of a protein boost in association with the DNA or Pox vectors to improve the magnitude and the quality of humoral responses 2019). In the HVTN 096 study [32], we have observed a high prevalence of HIV humoral response following two Nyvac boost. However, it is important to underscore that in this study a different DNA vaccine has been used as well different HIV antigens were expressed in both DNA and Nyvac vaccines as compared to vaccines tested in this current EV03/ANRS Vac 20 trial. We show here that2 DNA-C prime followed by 2 NYVAC-C boosts appeared to stimulate IgG responses to the various consensus gp140 antigens (group M, clade B, clade C) in more subjects compared to the 3 DNA-C prime and 1 NYVAC-C boost, suggesting that 2 boosts with a live virus vector may be more effective at eliciting IgG binding antibodies compared to only 1 boost. Env IgG3, a correlate of decreased HIV-1 risk in the RV144 [33] and HVTN 505 trials [34] was elicited in <20% of vaccinees. There was a higher magnitude of Env IgG3 in the 3xDNA compared to the 2xDNA group; however these data are from a small number of responders. Circulating Env IgA was previously shown to directly correlate with HIV-1 risk [35] and influence the correlation of Ab Fc effector functions with HIV-1 risk [34]. Notably, the vaccine regimens tested here elicited no significant circulating Env IgA by either vaccine regimen. Further studies are needed to determine whether these regimens elicited differences in antibody Fc effector functions associated with protection in NHP studies (reviewed in [36]) and/or correlated with HIV-1 risk in human clinical trials (reviewed in [29]). However, the recent negative results from HVTN 702 suggest that non-neutralizing responses may not be effective in high risk populations as compared to the low risk population involved in the RV144 trial. In depth analysis of the humoral responses of the HVTN 702 trial would provide further clarification on the real importance of the immune correlates of protection observed in the RV144 trial.

Our major focus in this study was to reinforce T-cell responses by optimizing the prime-boost regimen. The importance of T-cell response in the protection and/or the control of HIV replication after infection [37, 38] is supported by the results of several preclinical studies. We show here that the modulation of the prime strategy, without modifying the route or the dose of administration, or the combination of vectors, may influence the quality of the responses. One advantage of this strategy is the lack of *in vivo* responses against the vector itself allowing the administration of three DNA-C prime.

Large phase IIb/III studies under development are logically built on the promising results of the Thai trial and thus essentially focused on the poxvirus prime followed by poxvirus plus protein boosts. However, our results support the preclinical data demonstrating the potential of DNA as a prime capable not only of improving immune responses but also of imprinting the long-term responses to boost vaccines. Combination regimens testing the co-administration of DNA-C and Env protein with and without Pox vectors are options that should also be considered in the design of future prophylactic or therapeutic vaccine studies.

## Materials and methods

### Ethics statement

The study protocol was approved by the Comité de Protection des Personnes–Ile-de-France IX for France, Commission d’éthique de la recherche clinique (Université de Lausanne) for Switzerland, St Mary’s LREC (Local Research Ethics Committee) and GTAC (Gene Therapy Advisory Committee) for the UK, and Ethics Committee of Universität Regensburg for Germany.

Regulatory approval was also obtained from the competent authorities in all countries, AFSSAPS (Agence Française de Sécurité Sanitaire des Produits de Santé) for France, Swissmedic (Swiss Agency for Therapeutic Products) for Switzerland, MHRA (Medicines and Healthcare Products Regulatory Agency) for the UK, and PEI (Paul-Ehrlich-Institut) for Germany.

### Vaccines

The plasmid DNA vector and recombinant NYVAC-C were manufactured according to Good Manufacturing Practice. Both express the same HIV genes CRF_07 BC gag, pol, nef and envgp120 genes. The DNA-C vaccine was produced by Cobra Biomanufacturing (Keele, UK) and prepared for the trial in vials by ProPharma, all according to Good Manufacturing Practice. The presentation was in liquid form, with an extractable volume of 2 ml in 5 ml vials stored at −20°C or lower. The appearance was clear and the composition per milliliter as follows: 1.0 mg DNA-C, 1.57 mg Tris-HCl buffer, 0.372 mg EDTA, 9mg NaCl, and water to 1 ml. Prior to use, the vials were thawed at room temperature and the DNA-C vaccine was administered by intramuscular injection of 2ml into each vastus lateralis muscle (total of 4ml per vaccination) by a member of the local study team. The vaccine NYVAC-C (vP2010), lot Z141, was produced by Transgene SA (Strasbourg, France) and formulated with a potency of 10^7^ pfu/ml. It was presented in a liquid form, in a volume of 1ml in single dose 2ml glass ampoules, which were stored at −20°C at the least. Prior to use, each vial was thawed at room temperature and the vaccine was administered by intramuscular injection into the deltoid muscle of the non-dominant arm by a member of the local study team.

### Study design

**Fig 7:**
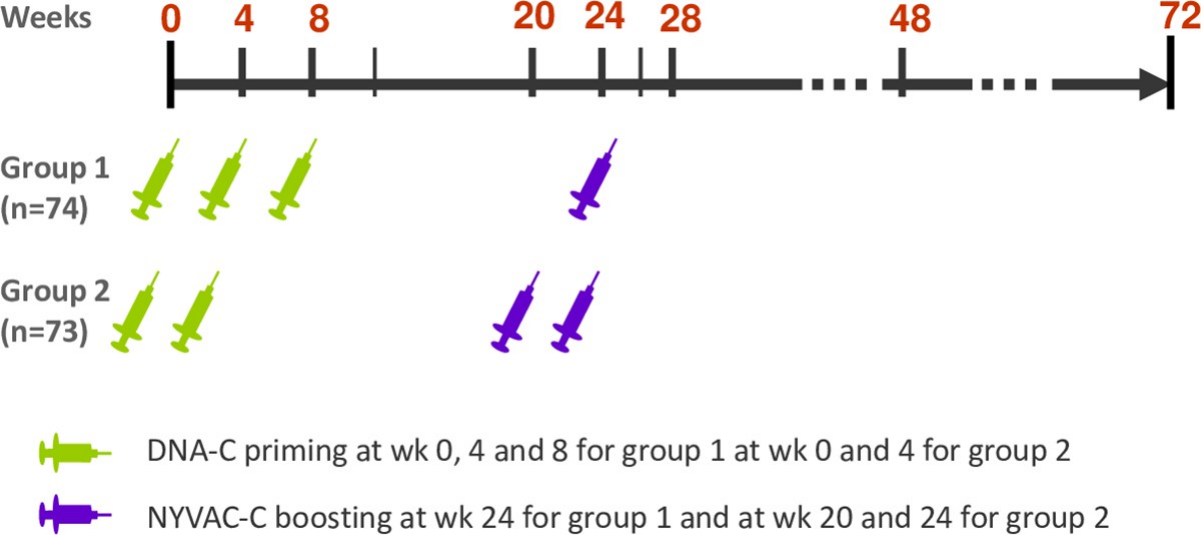
Trial design, EV03/ANRS VAC20 Phase I/II Trial. Schematic representation of EV03 study design: three injections of DNA (at weeks 0, 4 and 8) plus one NYVAC (at week 24) or two DNA (weeks 0, 4) plus two NYVAC (weeks 20, 24) were administered in 3xDNA and 2xDNA groups, respectively.

The EV03/ANRS VAC20 (ClinicalTrials.gov number NCT00490074) was a randomized Phase I/II trial conducted in 8 sites: five in France (three in Paris, one in Marseille and one in Toulouse), one in each in Switzerland (Centre Hospitalier Universitaire Vaudois, CHUV Lausanne), England (St Mary’s Hospital, Imperial College, London) and Germany (University of Regensburg). This trial was co-sponsored by the EuroVacc Foundation (EVF) & the French National Institute for Health and Medical Research-France Recherche Nord & Sud Sida-HIV Hépatites (Inserm-ANRS). Participants were randomized to receive either three administrations of DNA (at weeks 0, 4 and 8) plus one NYVAC (at week 24) (3xDNA group) or two DNAs (weeks 0, 4) plus two boosts of NYVAC (weeks 20, 24) (2xDNA group), stratified by geographical region and sex (Fig 7). Participants and clinical investigators were not blinded but laboratory personnel undertaking and interpreting the assays were. Participants attended clinic at screening (within 6 weeks of randomization), at immunization time points, and, for evaluation of solicited adverse events, once within the first 3 days and again 1 week after each immunization time point.

### Study participants

All volunteers provided written consent to participate in the trial, before screening in ANRS sites (France) and in Klinikum-UREG (Regensburg, Germany), or before screening and before enrollment at St Mary’s (London, UK) and at CHUV (Lausanne, Switzerland). Volunteers were eligible if aged between 18 and 55 years; at low risk of HIV infection; if they had no history of injecting drug use in the previous ten years, no gonorrhea or syphilis in the last six months, no high risk partner (e.g. injecting drug use, HIV positive partner) either currently or within the past six months, no unprotected anal intercourse in the last six months, outside a relationship with a regular partner known to be HIV negative, no unprotected vaginal intercourse in the last six months outside a relationship with a regular known/presumed HIV negative partner; they were willing to undergo a HIV test and a genital infection screen; using an effective form of contraception; and available for the duration of the study. Exclusion criteria were: clinically relevant abnormality including a history of severe local or general reaction to a licensed vaccine or recent receipt of a live attenuated vaccine, blood products or immunoglobulin; positive serology for HIV, hepatitis B surface antigen, hepatitis C antibody or serology compatible with active syphilis; positive DNA/ANA antibodies at a clinically relevant titer; or mild elevation in laboratory parameters other than unconjugated hyperbilirubinemia.

Volunteers were recruited through the ANRS network of volunteers or through advertising in the hospitals, universities, colleges, newspapers, magazines and on the radio and were given a telephone number to contact. They were provided with further information about the study and asked to complete a short interview (by telephone or in person) to assess their suitability. They were then given or sent an information sheet in an appropriate language about the trial. Recruitment strategies varied between centres but all the information provided to volunteers was standardised and information was recorded according to standardised procedures.

### Safety evaluation

Data on local and systemic events were solicited with specific questions during a period of at least 7 days following each immunization. Participants were given a diary card to assist in recall, and the nurses/physicians went through this with them at the next scheduled visit. Data on other clinical and laboratory events were collected with an open question at each visit and through routine scheduled investigations, respectively. Routine hematology and chemical pathology were performed at screening and twice after each immunization in accredited laboratories attached to each center. All events were graded in the same way as in the preceding EuroVacc trials [18, 19].

### Immunological evaluation

#### IFN-γ ELISPOT assay

The immunogenicity of vaccines was assessed by the quantification of CD4/CD8^+^ T-cell responses, i.e Spot Forming Units (SFUs), evaluated using an IFN-γ ELISPOT assay as described [19], against a panel of HIV overlapping peptides (15-mers with 11 amino acid overlap, n = 474) spanning the entire Gag/Pol/Nef polygene, and the Env clade C of HIV-1 97CN54, grouped in 8 pools (Gag1, Gag2, Gag/Pol, Pol1, Pol2, Nef, Env1, Env2). Assays were performed in a centralized laboratory (CHUV, Lausanne) on cryopreserved peripheral blood mononuclear cells (PBMC) at weeks 0, 4, 8, 12 (group 1), 20 (group 2), 24, 26, 28, 48 and 72. The assay was considered as valid if the negative control was lower than 50 SFU/10^6^ cells and the positive control (Staphylococcus Enterotoxin B, SEB) above 500 SFU/10^6^ cells.

#### Intracellular Cytokine Staining (ICS) Assay

To assess antigen-specific T-cell responses, ICS assay was performed in a centralized laboratory (MIC-VRI, Creteil, France) on cryopreserved PBMC of IFN-γ ELISPOT responders to Env pools at week 26/28. PBMC were rested overnight and then stimulated (6h, 37°C, 5% CO_2_) with the aforementioned peptide pools (1μg/ml for each peptide) in the presence of anti-CD28 antibody (1μg/ml) and Golgi Plug (1μl/ml) (BD Biosciences, Le Pont de Claix, France). SEB stimulation (100 ng/ml SEB; Sigma Aldrich, Saint Quentin Fallavier, France) served as a positive control. After stimulation, cells were stained for dead cells with an amine-reactive dye (LIVE/DEAD Aqua, Invitrogen, Life Technologies, Saint Aubin, France) for 20 minutes at room temperature, washed and labeled with fluorochrome-conjugated monoclonal antibodies (anti-CD3 Alexa700, anti-CD4 PE-CF594, and anti-CD8 PacBlue; all from BD Biosciences) for 15 min at room temperature. After fixation and permeabilization using Cytofix/Cytoperm kit (BD Biosciences) for 20 min and staining with anti-IFN-γ FITC, -TNF-α PE-Cy7 and -IL-2 APC (all BD Biosciences) for 20 minutes at room temperature, PBMCs were re-suspended in BD CellFIX (BD Biosciences) and stored at 4°C until analysis. Data were acquired on a LSRII flow cytometer (Becton Dickinson) 3 lasers (405, 488, 640 nm). At least 250,000 events gated on CD3^+^ were collected and analyzed with FlowJo software, version 9.3.3. Responses were defined as positive if the % cytokine+ in CD4^+^ or CD8^+^ T cells was > 0.03% and 3-fold or greater above background.

#### Multiplex measurements

Among the volunteers selected for ICS, functional profiles were further characterized before (week 0) and after vaccination (week 26/28) by multiplex measurements in the central laboratory of MIC-VRI Créteil: PBMC were stimulated with 6 HIV peptide pools (Env1, Env2, Pol1, Pol2, Gag1, Gag2, 1μg/ml each) and supernatants were collected at day 2 and day 5. As negative and positive controls, cells were cultured in medium alone or in the presence of the superantigen, SEB, respectively. The following analytes were analyzed in a 9-Plex kit including IFN-γ, IL-2, IL-5, IL-10, IL-13, TNF-α, Granzyme A, Granzyme B and Perforin (MILLIPLEX MAP kit, human CD8^+^ T cell magnetic bead panel, Millipore, Molsheim, France) and a 3-Plex for IL-17A, IL-21 and IP-10 (Procarta kit, Affymetrix/eBioscience, Paris, France) according to the manufacturer’s instructions. Median fluorescence intensity for each sample was measured using the Bio-Plex 200 system (Bio-Rad, Marnes-la-Coquette, France). The Bio-Plex Manager software version 6.0 (Bio-Rad, Marnes-la-Coquette, France), incorporating a weighted five-parameter logistic curve-fitting method, was used to calculate sample cytokine concentrations. Proportions of right/left−censored data (concentration data out of range (OOR) due to lower detection/upper saturation limits) were evaluated and statistical tests were performed as follows: if more than 70% of paired data were OOR at both time points, no statistical test was performed; if 50% of the data were OOR for at least one time point, McNemar test was performed; in the other cases, paired Wilcoxon signed−rank test was used. Participants with paired data OOR were excluded from the test. Data OOR at only one time was imputed at the plate−specific OOR threshold value. To account for test multiplicity and dependency among statistical tests of different cytokines, we used dependent FDR−Adjusted [39]. Adjusted P−values < 0.05 were considered significant.

#### Neutralizing antibody assays

Neutralizing antibodies were measured as a function of reductions in luciferase (Luc) reporter gene expression after a single round of infection in TZM-bl cells [40]. TZM-bl cells (also called JC57BL-13) were obtained from the NIH AIDS Research and Reference Reagent Program, as contributed by John Kappes and Xiaoyun Wu. Briefly, a pre-titrated dose of virus was incubated with serial 3-fold dilutions of test sample (1:20 starting dilution, 8 dilutions total) in duplicate in a total volume of 150 μl for 1 hr at 37°C in 96-well flat-bottom culture plates. Freshly trypsinized cells (10,000 cells in 100 μl of growth medium containing 75 μg/ml DEAE dextran) were added to each well. One set of 8 control wells received cells + virus (virus control) and another set received cells only (background control). After 48 hours of incubation, 100 μl of cells was transferred to a 96-well black solid plate (Costar) for measurements of luminescence using the Britelite Luminescence Reporter Gene Assay System (PerkinElmer Life Sciences). Assay stocks of molecularly cloned Env-pseudotyped viruses were prepared by transfection in 293T/17 cells (American Type Culture Collection) and titrated in TZM-bl cells as described [40]. This assay has been formally optimized and validated [41] and was performed in compliance with Good Clinical Laboratory Practices, including participation in a formal proficiency testing program [42]. Additional information on the assay and all supporting protocols may be found at http://www.hiv.lanl.gov/content/nab-reference-strains/html/home.htm.

#### Binding antibody assays

HIV-1 specific antibodies to HIV-1 gp120/gp140 proteins and V1-V2 scaffolds were measured in 121 subjects overall (with 56 in the 3xDNA and 65 in the 2xDNA groups) by a custom HIV-1 binding antibody multiplex assay as previously described [28, 33, 43, 44]. All assays were run under GCLP compliant conditions, including tracking of positive controls by Levy-Jennings charts using 21CFR Part 11 compliant software. Polystyrene beads were covalently coupled to A1.Con env 03 gp140 CF, B.con env 03 gp140 CF, C.con env03 gp140 CF, and Con S gp140 CFI (Haynes Lab, Duke Human Vaccine Institute, Durham, NC) as well as CN54 gp140 (Polymun Scientific, Klosterneuburg, Austria). Beads were incubated with serum at a 1:20 dilution for IgG and IgG3 assays. Serum for the IgA assays was first depleted of IgG and then incubated at a 1:10 dilution with coupled beads. Positive controls included a HIVIG titration, an IgA control, and IgG3 standard curve titrations. Negative controls included in every assay were blank beads, HIV-1 negative sera, and baseline (pre-vaccination) samples. To control for antigen performance, we used the preset criteria that the positive control titer included on each assay had to be within +/- 3 standard deviations of the mean for each antigen (tracked with a Levy-Jennings plot with preset acceptance of titer (calculated with a four-parameter logistic equation, LabKey Software). HIV-specific Ab isotypes were detected with mouse anti-human IgG (Southern Biotech, goat anti-human IgA (Jackson Immunoresearch) or mouse anti-human IgG3 (Calbiochem) conjugated to biotin, at 4 μg/ml, followed by washing and incubation with streptavidin-PE (BD Pharmingen). Antibody measurements were acquired on a Bio-Plex 200 instrument (Bio-Rad, Hercules, CA) using 21CFR Part 11 compliant software and the readout is in MFI. The preset assay criteria for sample reporting were: coefficient of variation (CV) per duplicate values for each sample were <15% and >100 beads counted per sample. The positivity of a response was defined based on background-adjusted blank-subtracted MFI values (net MFI) where the net MFI threshold was calculated using the 95^th^ percentile of the baseline measurements for each antigen and isotype. The standard definition to identify positive responses was defined as:

1. Net MFI ≥ antigen specific threshold, the antigen specific threshold was calculated based on the 95^*th*^ percentile of net MFI of the baseline samples for each isotype and antigen. The threshold should be no less than 100 MFI.
2. Net MFI *>* 3 × Net MFI*baseline
3. MFI-bkgd *>* 3 × (MFI-bkgd) baseline.

#### Vaccinia-specific antibody levels

Serum binding antibodies were detected using ELISA with cell-associated vaccinia NYCBH grown in BSC40 cells, UV-inactivated, and coated at 1:1,5000. Baseline sera were tested from 1:100 to 1:24,300 serial dilutions in duplicate. Each plate included Vaccinia Immune Globulin (VIG) from 167 to 0.69 ng/ml as internal control. Antibody levels were calculated from specimen and on-plate VIG standard curves. Neutralizing antibodies (nAb) were studied using β galactosidase-vaccinia and HeLa cells. In brief, sera serially 3-fold diluted from 1:20 to 1:540 or VIG control were incubated with vaccinia for one hour at 37 ^o^C and added to freshly plated HeLa cells in replicate 96-well U bottom wells. At 18 hours, cell lysates were assayed for β galactosidase activity using CPRG substrate (Roche, Florham Park, New Jersey, USA) with standard conditions, and activity detected on a plate reader at 575 nM. Results are expressed as the inverse dilution of sera required to inhibit 50% β galactosidase activity. Assays in which the VIG potency fell outside a pre-determined range were repeated.

### Study endpoints

All clinical events and routine laboratory data were included in the safety analysis. The adverse events and reactions were graded according to the same toxicity table used in the preceding EuroVacc trials [18, 19]. The primary safety endpoints were: grade 3 or above local adverse event (pain, cutaneous reactions including induration); grade 3 or above systemic adverse event (temperature, chills, headache, nausea, vomiting, malaise, and myalgia); grade 3 or above other clinical or laboratory adverse event confirmed at examination or on repeat testing, respectively; any event attributable to vaccine leading to discontinuation of the immunisation regimen.

The primary immunogenicity endpoint was defined as the presence of CD4/CD8^+^ T cell responses to Env plus at least one of the Gag, Pol, Nef peptide pools at weeks 26 or 28.

An ELISPOT response to individual peptide pools was considered as positive if the response was ≥ 4-fold the negative control and ≥ 55 SFU/10^6^ cells (as in EV01 and EV02 trials). In both EV01 and EV02 trials, a small proportion (<5%) of participants showed persistent IFN-γ ELISPOT reactivity to peptide pools prior to vaccination and subsequently across several timepoints. Participants with IFN-γ ELISPOT reactivity to peptide pools prior to immunisation were considered to have non-specific cross-reactive responses to the peptide pools, and subsequent responses to these pools were excluded from the immunogenicity analysis.

As a secondary endpoint, the magnitude of the ELISPOT response was calculated as the sum of SFUs of positive responses for each of the different HIV proteins at weeks 26 or 28. The durability of the responses was assessed at weeks 48 and 72.

### Statistical methods

All safety endpoints were graded by the clinical investigators. Any queries about grade and relationship to study product that could not be resolved by the Operational Sub-Group were referred to the Trial Coordinating Committee for a final decision. For the primary analysis of safety endpoints, results were expressed as a proportion and the two groups compared using Fisher’s exact test.

The two groups, 2xDNA and 3xDNA, were compared in terms of the proportion responding to Env plus Gag/Pol or Nef using a Chi-square test. As a secondary analysis, a multinomial logistic regression model was fitted to compare the proportion of participants at weeks 26 and 28 in the following categories: i) no response; ii) response to Env only or iii) response Gag/Pol/Nef (± Env). This provided a global test of the null hypothesis that the proportion in the three response categories in 3xDNA is the same as in 2xDNA. If the global test was significant, the two groups could then be compared in terms of the proportion responding to Env alone and in terms of the proportion responding to Gag/Pol/Nef (relative to no response). The magnitude of the responses was compared between randomized groups using non-parametric statistical tests (Mann-Whitney test). Logistic and linear regression analyses were used to analyse possible predictors of the primary endpoint and the response magnitude (log_10_-transformed), respectively, examining randomisation group, sex, age, geographical area and presence of antibodies against vaccinia.

For the binding antibody response analysis, response rates were calculated for each antigen and group with corresponding 95% Wilson score confidence intervals [45]. To assess if the groups had different response rates, testing was performed using Barnard’s exact test (two-sided, alpha = 0.05) for each isotype and antigen ([46] and [47]). To assess if the magnitude of response differs between groups, testing was performed on responders using Wilcoxon rank-sum test (two-sided, alpha = 0.05) for each isotype and antigen, if three responders are present in each group.

The sample size estimation was based on the following considerations: among 20 participants assigned to 2xDNA in EV02 [18], 30% responded to Env plus at least one of Gag, Pol or Nef. Assuming the same proportion in the 2xDNA group (control group) in EV03/ANRS VAC20, then 63 participants per group would be able to provide 90% power to detect a 30% difference between 2xDNA and 3xDNA (30% vs 60%) in the proportion of responders to Env plus Gag/Pol/Nef. An absolute difference of 25% (30% vs 55%) would be detected with 80% power. It was proposed to recruit 70 to each group, 140 in total, to allow for loss to follow-up, and failure to complete the allocated regimen for other reasons.

## Supporting information

S1 Table Number (%) of participants with local and systemic reactions following DNA and NYVAC immunisations, EV03/ANRS VAC20 Phase I/II Trial. (XLSX)

S2 Table Proportion of responders to individual peptide pools at the primary time points (week 26/28) and at weeks 48 and 72, EV03/ANRS VAC20 Phase I/II Trial. (XLSX)

S1 Fig Binding Antibody Responses (IgG responses to cross-clade consensus gp140 antigens), EV03/ANRS VAC20 Phase I/II Trial. Response rates with 95% confidence intervals (top) and magnitude of response (bottom) at week 26 for IgG consensus antigens, colored by group, with open triangles for non-responders and close circles for responders.
(TIF)

S2 Fig Binding Antibody Responses (correlation of IgG and IgA responses), EV03/ANRS VAC20 Phase I/II Trial. Correlation between IgG and IgA net MFI (IgG Responders Only) colored by group and the shape denotes the type of response, for week 26 and antigens A1.Con env03 140 CF and CN54 gp140. Spearman estimates with p values are shown overall (pooled) and by group.
(TIF)

S1 Protocol A phase I/II trial to compare the immunogenicity and safety of 3 DNA C prime followed by 1 NYVAC C boost to 2 DNA C prime followed by 2 NYVAC C boost (EuroVacc 03/ANRS Vac20). (PDF)

S1 Text CONSORT 2010 checklist. (DOC)
