# Supplementary figures and images for: Optimal priming of poxvirus vector (NYVAC)-based HIV vaccine regimens for T cell responses requires three DNA injections. Results of the randomized multicentre EV03/ANRS VAC20 Phase I/II Trial
Source: PLoS Pathog. 2020 Jun 26;16(6):e1008522. doi: 10.1371/journal.ppat.1008522 (PMC7319597; doi:10.1371/journal.ppat.1008522)

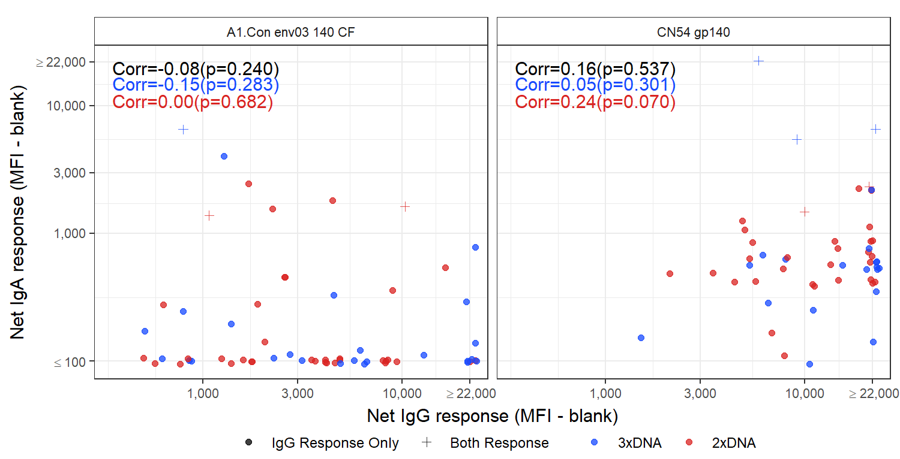

Supplement: S2 Fig — Correlation between IgG and IgA net MFI (IgG Responders Only) colored by group and the shape denotes the type of response, for week 26 and antigens A1.Con env03 140 CF and CN54 gp140. Spearman estimates with p values are shown overall (pooled) and by group. (TIF) [file ppat.1008522.s004.tif]
