# Supplementary material for: Optimal priming of poxvirus vector (NYVAC)-based HIV vaccine regimens for T cell responses requires three DNA injections. Results of the randomized multicentre EV03/ANRS VAC20 Phase I/II Trial
Source: PLoS Pathog. 2020 Jun 26;16(6):e1008522. doi: 10.1371/journal.ppat.1008522 (PMC7319597; doi:10.1371/journal.ppat.1008522)
Supplement: S1 Protocol — (PDF) [file ppat.1008522.s005.pdf]

EudraCT number – 2006-006141-13

ISRCTN number – ISRCTN20946776

EuroVacc 03/ANRS Vac 20

A phase I/II trial to compare the immunogenicity and safety of 3 DNA C prime followed by 1 NYVAC C boost to 2 DNA C prime followed by 2 NYVAC C boost

The EuroVacc Foundation will act as Sponsor for the trial in Eurovacc centres
The ANRS will act as sponsor for the trial in France (ANRS Centres)
The ANRS will act as the legal representative of the EuroVacc Foundation in the EU member states

**Version**	**Date**	**Reason for Update**
2.0	25th October 2006	Submitted by CHUV to local Ethics Committee
2.0	08th November 2006	Eudract number added<br>Note of clarification added to section 1.5 page 18 at request of Paul Ehrlich Institute
2.1	28th November 2006	Inclusion criteria: 54 weeks updated to 78 weeks.<br>Updates from Lausanne ethics committee comments.<br>Section 1.5.1 page 17 “excluding events unrelated to vaccine” deleted.<br>Section 7.1 page 28 storage time added.

2.2	18th January 2007	Front pages and section 9 : One immunology laboratory in France (instead of 2). Clarification of the Sponsors' responsibilities for the EuroVacc/ANRS.trial centres.<br>Sections II, III and 8: Primary outcome and analyses simplified from 3 categories to 2 after review of 5,000 simulations using 3 categories by Professors Babiker, Chene (Statisticians), Levy and Pantaleo (Immunologists). Three categories to become secondary analysis.<br>Section 1.2.2: "Start of enrolment and duration of the study" added.<br>Section 1.3: Additional inclusion criteria added (for French volunteers only).<br>Section 1.4: NYVAC will be packaged in ampoules and should be stored at -20ºC<br>Sections 1.5.2, 2.6 and 4.2.1: "Antibody responses" replaced by "Sera Sampling"; assays will be determined at a later stage but prior to unblinding of laboratory personnel.<br>Section 2.6: Study schedule updated.<br>Section 7.1: "Return of SAE form" replaced by "Notification of SAEs". "within 2 or 10 working days…" replaced by "as soon as they be aware of the event"<br>Appendices 1 and 2 updated to reflect version 2.2. No changes to the Participant Information Sheet required since the version of 25th October 2006.
3.0	18 April 2008	Cover page: ISRCTN number added<br>Summary of the trial (p.14) and section 1.5: definition of the primary immunogenicity endpoint clarified according to TCC recommendations, based on the definition used in previous EV02 vaccine trial.<br>Summary of the trial (p.15) and section 7.7: DSMC members updated<br>Section1.3: Note of clarification added to exclusion criterion "grade 1 or above routine laboratory parameters" related to bilirubin parameter<br>Section 1.4: NYVAC-C and DNA-C can be stored at -20°C or below. Quantity of DNA in DNA-C vaccine is 1 mg.<br>Section 2.6: Study schedule updated (windows for visits added, eligibility evaluation deleted at week 20 (typing error), comment "f" added, cell storage for HLA shifted from week 0 to week 4)<br>Section 4.1.1 and appendix 4, Cutaneous vaccine chart (p.50): Precision on induration definition added (should read "induration (hardened swelling)").<br>Section 4.1.4: Clarification of immunological parameters to be undertaken for safety assessment<br>Section 5.3: Precision on the reporting of SAEs (multi-disciplinary review)<br>Section 7.6: TCC members updated<br>Section 7.7.2: Precision on indications for discontinuation of the trial<br>Appendix 4, Laboratory parameters chart (p.47): white blood count grade 4 should read "≥30.0" instead of ">30.0"

The trial will be conducted at some or all of the following centres, under the supervision of the named site Investigators, with Professor Pantaleo acting as Chief Investigator for the EuroVacc centres and Professor Levy as Chief Investigator for the ANRS centres:

**EuroVacc Centres**		
Professor Giuseppe Pantaleo	Division of Immunology and Allergy<br>Centre Hospitalier Universitaire Vaudois<br>1011 Lausanne<br>Switzerland	giuseppe.pantaleo@chuv.ch<br>Tel: 00 41 21 3141071<br>Fax: 00 41 21 3141070
Professor Bernd Salzberger	Klinikum für Innere Medizin<br>Klinikum Universität Regensburg<br>93042 Regensburg<br>Germany	bernd.salzberger@klinik.uni-r.de<br>Tel: 00 49 941 944 7143<br>Fax: 00 49 941 944 7144
Professor Jonathan Weber	Department of GU Medicine and Communicable Diseases<br>Imperial College Science, Technology and Medicine<br>St Mary's Hospital<br>Praed Street<br>London W2 1NY<br>UK	j.weber@imperial.ac.uk<br>Tel: 00 44 207 594 3901<br>Fax: 00 44 207 594 3643
**It is the intention to expand the EuroVacc centres to include a South African site.**		
**ANRS Centres**		
Professor Yves Lévy/<br>Dr Jean Daniel Lelièvre	Service d'Immunologie Clinique<br>Hôpital Henri Mondor<br>51, avenue du Maréchal de Tassigny<br>94010 Créteil cedex<br>France	yves.levy@hmn.ap-hop-paris.fr<br>Tel: 33 (0) 1 49 81 24 55<br>Fax: 33 (0) 1 49 81 24 69<br>lelievre@im3.inserm.fr<br>Tel: 33 (0) 1 49 81 37 19<br>Fax: 33 (0) 1 49 81 37 09
Dr Odile Launay	CIC de vaccinologie Cochin-Pasteur ,<br>Service de Médecine interne<br>Hôpital Cochin,<br>27 rue du fg. Saint Jacques,<br>75679 Paris Cedex 14<br>France	odile.launay@cch.ap-hop-paris.fr<br>Tel: 33 (0) 1 58 41 28 58<br>Fax: 33 (0) 1 40 46 93 08
Professor Gilles Pialoux	Service des Maladies Infectieuses et Tropicales,<br>Hôpital Tenon, Paris<br>4, rue de la Chine<br>75970 Paris cedex 20<br>France	gilles.pialoux@tnn.ap-hop-paris.fr<br>Tel: 33 (0) 1 56 01 74 17<br>Fax: 33 (0) 1 56 01 74 18
Dr Lise Cuzin	Service des Maladies Infectieuses et Tropicales,<br>Hôpital Purpan,<br>Place du Dr Baylac<br>31059 Toulouse Cedex 09<br>France	cuzin.l@chu-toulouse.fr<br>Tel: 33 (0) 5 61 77 90 43<br>Fax: 33 (0) 5 61 77 21 38

Dr Benedicte Bonnet	Services des Maladies Infectieuses et Tropicales,<br>Hôpital de Nantes<br>Hôtel Dieu, aile ouest<br>Place Alexis Ricordeau<br>44 093 Nantes cedex 01<br>France	benedicte.bonnet@chu-nantes.fr<br>Tel: 33 (0) 2 40 08 31 12<br>Fax: 33 (0) 2 40 08 33 72
Dr Isabelle Poizot-Martin	Service d'Hématologie, Hôpital Ste Marguerite,<br>270, Bd Sainte Marguerite<br>13009 Marseille<br>France	isabelle.poizot@ap-hm.fr<br>Tel: 33 (0) 4 91 74 61 63<br>Fax: 33 (04 91 74 50 69

The trial will be jointly coordinated by the INSERM U593/Clinical Trials Unit (ANRS centres) and the MRC Clinical Trials Unit (EuroVacc centres) by the following Investigators:

Professor Genevieve Chene	INSERM U593/Clinical Trials Unit<br>ISPED – 146 rue Léo-Saignat<br>33076 Bordeaux cedex, France	genevieve.chene@isped.u-bordeaux2.fr<br>Tel : 00 33 5 5757 13 92<br>Fax : 00 33 5 5757 11 72
Professor Abdel Babiker<br><br>Dr Sheena McCormack<br>(EuroVacc Medical Expert)	MRC Clinical Trials Unit<br>222 Euston Road<br>London NW1 2DA, UK	a.babiker@ctu.mrc.ac.uk<br>Tel: 00 44 207 670 4703/32<br>s.mccormack@ctu.mrc.ac.uk<br>Tel: 00 44 207 670 4708/14<br>Fax: 00 44 207 670 4815

Immunogenicity endpoint assessments will be conducted in two laboratories, overseen by the following Investigators:

Professor Giuseppe Pantaleo<br>Dr Alexandre Harari<br>(ELISPOT assays)	Division of Immunology and Allergy<br>Centre Hospitalier Universitaire Vaudois<br>1011 Lausanne, Switzerland	Alexandre.Harari@chuv.hospvd.ch<br>Tel: 00 41 21 314 1069<br>Fax: 00 41 21 314 1070
Professor Yves Levy<br>(Flow cytometry)	Equipe INSERM U841<br>Faculté de Médecine de Créteil<br>8, rue du géneral Sarrail<br>94010 Créteil	yves.levy@hmn.aphp.fr<br>Tel: 00 33 1 4981 2455<br>Fax: 00 33 1 4981 3709

NYVAC C will be provided by:

EuroVacc Foundation
Professor Peter Liljestrom
Rue de la Grotte 6
1003 Lausanne
Switzerland
Peter.Liljestrom@mtc.ki.se

DNA C will be provided by:

University of Regensburg
Professor Hans Wolf hans.wolf@klinik.uni-regensburg.de
00 49 941 944 6401
Professor Ralf Wagner Ralf.Wagner@klinik.uni-regensburg.de
00 49 941 944 6452
Institute for Medical Microbiology and Hygiene
Franz-Josef-Strauss-Allee 11
D-93053 Regensburg
Germany
Fax: 00 49 941 944 6402

The trial will be sponsored :
- in the EuroVacc centres by

The EuroVacc Foundation
Professor Peter Liljestrom
Rue de la Grotte 6
1003 Lausanne
Switzerland
Peter.Liljestrom@mtc.ki.se

The trial will be sponsored :
- in the ANRS Centres (France) by

The ANRS
Professor Jean François Delfraissy jf.delfraissy@anrs.fr
101, Rue de Tolbiac 75013 Paris 00 33 1 53 94 60 23
France Fax : 00 33 1 53 94 60 01

The Legal Representative of the EuroVacc Foundation in the EU member states will be the ANRS

Principal investigator for EuroVacc centres

Professor Giuseppe Pantaleo giuseppe.pantaleo@chuv.ch
Division of Immunology and Allergy Tel: 00 41 21 3141071
Centre Hospitalier Universitaire Vaudois Fax: 00 41 21 3141070
1011 Lausanne
Switzerland

Principal investigator for ANRS centres

Professor Yves Lévy yves.levy@hmn.ap-hop-paris.fr
Service d'Immunologie Clinique Tel: 33 (0) 1 49 81 24 55
Hôpital Henri Mondor Fax: 33 (0) 1 49 81 24 69
51, avenue du Maréchal de Tassigny
94010 Créteil cedex
France

The signatures below constitute their approval of this protocol and provides assurance that this study will be conducted in their clinical centre(s) according to the stipulations contained within this document, including the statements of confidentiality, provision of indemnity for clinical staff employed by the institution, and good clinical practice. The signature of Professor Peter Liljestrom on behalf of the EuroVacc Foundation and Professor Jean François Delfraissy on behalf of the ANRS, provides assurance of the Sponsors' approval of the protocol including statements about provision of a policy to provide cover for clinical trial liability other than clinical negligence and negligence during the conduct of the clinical trial.

Signed ________________________________ Date: _________
Site Investigator

Signed ________________________________ Date: _________
Professor Jean François Delfraissy (ANRS)

Signed ________________________________ Date: _________
Professor Peter Liljestrom (EuroVacc)

Signed ________________________________ Date: _________
Professor Giuseppe Pantaleo

Signed ________________________________ Date: _________
Professor Yves Lévy

Contents

I Introduction
II Summary of Trial
III Scientific questions to be addressed

1 General plan
- 1.1 Objectives
- 1.2 Design
- 1.3 Population
- 1.4 Trial products
- 1.5 Endpoints

2 Schedule of visits
- 2.1 Recruitment
- 2.2 Screening
- 2.3 Enrolment and trial entry (including immunisation)
- 2.4 Follow-up visits
- 2.5 Final visit (including in event of a discontinuation)
- 2.6 Study schedule

3 Procedures
- 3.1 Blood and urine collection
- 3.2 Clinical history and examination
- 3.3 Genital infection screen
- 3.4 Pre-HIV test screen and counselling
- 3.5 Safe sex counselling and condom provision
- 3.6 HIV related issues (see also section 6)
- 3.7 Discontinuation procedures (including withdrawal)
- 3.8 Unblinding procedure

4 Assessments
- 4.1 Safety assessments
- 4.2 Immunogenicity assessments
- 4.3 Other assessments

5 Adverse events
- 5.1 Definitions
- 5.2 Relationship to study product
- 5.3 Reporting adverse events
- 5.4 Clinical management

6 Management of HIV issues during and following the trial
- 6.1 HIV testing
- 6.2 HIV infection
- 6.3 Social discrimination as a result of a post-vaccine response

7 Management of the trial
- 7.1 Data management at the clinical centre
- 7.2 Data management in the immunology laboratories
- 7.3 Data management at the MRC Clinical Trials Unit & INSERM U593 CTU
- 7.4 Monitoring by MRC Clinical Trials Unit & INSERM U593 CTU
- 7.5 Data ownership
- 7.6 Trial Coordinating Committee (TCC)
- 7.7 Data and Safety Monitoring Committee (DSMC)

8 Statistical considerations
- 8.1 Sample size
- 8.2 Primary analysis
- 8.3 Secondary analysis
  - 8.3.1 Immunogenicity
  - 8.3.2 Safety

9 Confidentiality, ethics and responsibilities including indemnity
10 Publication
11 References

Appendices
1 Information sheet for all participants
2 Informed Consents for screening, enrolment and long term follow-up
3 Packaging and labelling
4 Grading of clinical and laboratory events
5 Diary Card

.I Introduction

(i) Epidemiology

Globally, by the end of 2005 40.3 million people were estimated to be infected with HIV (UNAIDS 2005). AIDS killed 2.4 million African people in 2005 and is now the fourth commonest cause of death worldwide. Over 90% of HIV infections occur in developing countries, with the majority of infections found in sub-Saharan Africa (25.8 million) and East, South & South-East Asia (8.27 million). Whilst initiatives to deliver antiretroviral therapy are underway, there remains an urgent need for preventative measures such as health education, treatment of sexually transmitted diseases, vaccines and topical microbicides, for several reasons. Firstly, in geographical areas where the prevalence is as high as 50% in ante-natal cohorts, treating all infected individuals will be beyond the scope of the public health services. Secondly, even in the presence of a comprehensive treatment service, sexual transmission continues and this is clearly demonstrated in Europe where the estimated incidence of HIV infection in homosexual men is around 1-2% per year.

(ii) Molecular epidemiology

Following systematic phylogeny [1], HIV isolates can now be classified into discrete genetic subtypes. Improved molecular epidemiological studies by the WHO (subsequently UNAIDS) Network for HIV Isolation and Characterisation, and other groups have lead to understanding of HIV subtype transmission worldwide. Subtype B has been responsible for the HIV epidemic in homosexual men and intravenous drug users worldwide, and most immunogens, laboratory adapted isolates, reagents and mapped epitopes belong to subtype B. In sub-Saharan Africa, India and China, areas where the incidence of new HIV infections is high, subtype B accounts for only a small minority of infections, and subtype C appears to be the most common infecting subtype [2]. The biological significance of these genetic subtypes is far from clear. Antibody-mediated neutralisation studies from several laboratories have all shown that the genetic subtypes do not correspond to neutralisation serotypes [3, 4, 5]. Studies of CTL reactivity from infected subjects and from vaccinees suggest that cross-subtype CTL reactivity is common [6, 7]. Cross-reactive CTL have been described following immunisation with canarypox prime regimens [8]. While the clinical relevance of these results remains unclear, the current vaccine development strategy continues to be based on the axiom that the immunogen should closely resemble the circulating strain. Nonetheless, it remains important to find ways to more rigorously assess cross-reactivity in Phase I/II HIV vaccine trials, as this may be a key determinant in selecting products to take forward to large scale field trials.

(iii) HIV vaccine strategies under investigation

There is a broad scientific consensus that a successful vaccine to prevent HIV-1 transmission must be able to elicit both HIV-specific T cells and neutralising antibodies [9,10]. These include: live attenuated vaccines; inactivated viruses with adjuvants; subunit vaccines with adjuvants; live-vector based vaccines; and DNA vaccines. Major concerns regarding safety issues have been raised for the use of live attenuated vaccines in humans [11]. The protective immunity generated in monkeys immunised with inactivated viruses with adjuvants is not virus-specific [12]. Subunit vaccines, such as highly purified recombinant monomeric HIV-1 envelope proteins elicit neither virus-specific CTL nor antibody responses that can neutralize primary patients isolates of HIV-1, even when adjuvanted with potent immunostimulants [13], and no protection was demonstrated in the one Phase III trial to date [14].

Recent studies, both in non human primates and in humans, have shown that DNA based vaccines in combination with live-vector based vaccines in prime-boost regimens are able to induce both CD4 and CD8 T cell responses. These prime-boost combinations induce powerful T cell responses as measured by IFN-γ ELISpot assay, polifunctional (IFN-γ plus IL-2) CD4 and CD8 T cell responses and durable responses (up to one year after the last

immunisation T cell responses are still detectable). The results have been generated in clinical trials evaluating DNA prime plus Ad5 boost or pox vector (NYVAC) boost performed within the VRC and EuroVacc vaccine programmes, respectively.

The best studied vaccine vectors in humans are the pox viruses. Vaccinia virus engineered with HIV-1 genes have been shown to induce virus-specific cellular and humoral immune responses in immunized macaques and protection against simian immunodeficiency virus (SIV) infection when immunization with such constructs have been followed with boosting by recombinant proteins [8]. However, due to the development of life-threatening disseminated vaccinia infections in immunosuppressed individuals [15], there is a reluctance to use vaccinia virus as a vector system in large human trials, where unsuspected HIV infection may occur. Particular attention has been focused on pox viruses with limited in vivo replicative capacity and, therefore, limited pathogenicity, e.g. MVA or NYVAC, which have deletions of the genes associated with pathogenicity, or avian pox viruses, which do not complete an entire replication cycle in human cells but initiate protein synthesis and thus elicit immune responses. Clinical experience has been reported with a non-replicating pox virus construct which expresses several antigens derived from the malaria parasite *Plasmodium Falciparum* (NYVAC-Pf7). In this study, healthy volunteers received doses of 1 x 10^7 or 1 x 10^8 pfu with no adverse effects [16]. Several different vaccinia vectors expressing different HIV genes have been tested in Phase I trials in humans. These have appeared to be safe and well tolerated [17,18]. EuroVac 01 (EV01) recruited 24 healthy volunteers in London and Lausanne, 20 of whom were allocated to receive NYVAC HIV-C. There were no serious adverse events and indeed no grade 3 (severe) adverse events reported. EV02 has enrolled 40 participants randomised to receive two immunisations of 4mg DNA-C followed by two immunisations of NYVAC-C (n=23) or two immunisations of NYVAC-C alone (n=17). Early results of EV02 will be available before the commencement of EV03.

(iv) Study products

(iv)a DNA C

Derivation of product to be used in this trial

pORT- plasmid DNA vector system

Conventional DNA production processes usually require the use of antibiotics for selection purposes, and thus depend on the insertion of a gene that provides antibiotic resistance into the prophylactic or therapeutic DNA vaccine. However, both the presence of such selectable marker genes on plasmids, and the utilization of antibiotics during the growth of plasmids are undesirable and represent a significant and unnecessary risk to the volunteers' health.

Cobra's DNA-plasmid vector technology eliminates the need for both of these requirements which is particularly relevant given that the increase in AIDS has been associated with antibiotic resistant TB. Cobra has developed a simple robust manufacturing process for plasmid DNA, which does not use antibiotics at any stage of the process and does not require any selectable genes on the plasmid. This is achieved by moving the structural gene involved in selection from the plasmid to the chromosome of the host strain and then making the regulation of that gene dependant on a small non-coding regulatory sequence borne on the plasmid. The producer cell cannot survive without the presence of the plasmid and thus stabilises its production. The mechanism is based on a phenomenon known as repressor titration [19]. This DNA-vector system (pORT) was used to manufacture the clade A DNA for the HIV phase I clinical trials conducted in Oxford, London and Nairobi and the DNA malaria vaccine described above in section (iii).

pORT1a-HIV C clade

The HIV genes expressed in the recombinant vector are derived from the Chinese R5 CRF_07 HIV strain (97CN54) that has been isolated and characterized by the University of Regensburg [20]. This strain 97CN54 has been shown to be representative of the most prevalent virus strains circulating in China and India. All HIV genes have been optimised for

codon usage, RNA stability and nuclear export since it has recently been shown that humanisation of synthetic HIV gene codons allowed for an enhanced and REV/RRE-independent expression of env and gag-pol genes in mammalian cells. Genes were optimised for both safety and production efficiency.

The env gene has been designed to express the secreted gp120 form of the envelope proteins and contain an optimal synthetic leader sequence for enhanced expression. The gag, pol and nef genes were fused to express a Gag-Pol-Nef polyprotein. An artificial -1 frameshift introduced in the natural slippery sequence of the p7-p6 gene junction results in an in-frame Gag-Pol-Nef fusion protein due to the absence of ribosomal frameshift. An N-terminal Gly to Ala substitution in gag prevents the formation and release of virus-like particles from transfected cells. This strategy should allow for an equimolar production of Gag, Pol and Nef proteins and an enhanced MHC Class-I restricted presentation of their CTL epitopes.

For safety and regulatory reason, the packaging signal sequence has been removed; the protease active site mutated; the integrase gene deleted; and the reverse transcriptase gene disrupted by insertion of a scrambled nef gene at the 3' end of the DNA sequence coding for the RT active site known to be associated with an immunodominant CTL epitope. The nef gene has been dislocated by fusing its 5' half to its 3' half without losing its immunodominant CTL epitopes.

Safety, immunogenicity and challenge models of DNA vaccines in animals

In mice

So far, consequent application of DNA vaccines is limited by safety concerns as well as by its low immunogenicity and efficacy in primates. The heterologous expression of HIV group specific antigen (Gag) requires the presence of a 5´-untranslated region (UTR), including a RNA-packaging signal (ψ), as well as co-expression and binding of the Rev protein to the *Rev responsive element* (RRE). Substitution the REV/RRE-system by the Mason-Pfizer D-Type Retrovirus (MPMV) constitutive RNA transport element (CTE) resulted only in the presence of 5´-UTR (including ψ) in a high and Rev- independent $Pr55^{gag}$-expression. After integration of a functional intron upstream the *wtgag* gene acquired a REV/RRE and 5`UTR independent Gag expression. Finally the adaptation of the codon-usage to highly expressed mammalian genes (*syngag*) and subsequent alteration of the RNA-retention elements allowed (1) a REV/RRE/UTR-independent and enhanced gag expression, reduced (2) the homology between the vaccine construct and the *wtgag* sequence to the theoretical minimum and (3) increased the amount of immunostimulatory DNA-motifs.

The immunologic evaluation of different constructs was further tested in the BALB/c mouse model. The strongest immunostimulation was obtained after intramuscular immunisation with *synthetic Gag based DNA vaccine vector* and showed a significant T-helper-1 (Th-1) induced immune response accompanied by high titres of IgG2a antibodies, a significant IFN-γ secretion and a strong CTL response. The remainder of the Rev-independent constructs induced a $Pr55^{gag}$-specific Th-1-immune response that was reduced in all parameters by several logs as compared to the described *syngag* vector [21,22]. Furthermore, synthetic genes encoding full-length gag pol proved to abolish the opportunities for homologous recombination with wt-HIviral sequences to replication competent recombinants (RCRs) below detection level [23].

In rhesus macaques

Different DNA vaccine constructs, optimized for safety and efficacy by optimizing the genes for RNA stability, nuclear export and translation, were developed expressing SIVmac239 Gag and Tat in addition to envelope derivatives from $SHIV_{89.6P}$.

The immunogenicity and efficacy of such DNA vaccines were tested in a pathogenic $SHIV_{89.6P}$ rhesus macaque model. Groups of each four rhesus macaques were immunized either with a single antigen (Tat alone) or with a combination of regulatory and structural antigens (Tat/Env/Gag). Immunogens were provided following a prime-boost protocol. Priming was achieved by administering 500 µg plasmid DNA i.m./i.d., respectively. Primed responses were expanded by two booster immunizations injecting the corresponding set of purified antigens, respectively. To determine the high degree of protection afforded, animals were challenged with a series of high virus doses, ending in a final challenge of 50,000 MID_{50} of the pathogenic $SHIV_{89.6p}$ strain. Interestingly, the strongest immune responses in terms of neutralizing antibodies and specific cytokine secreting (IL-2, γIFN, IL-4; ELISPOT) were developed after immunisation with Tat alone, but these did not afford protection or containment of virus load. Immunisation with Tat/Env/Gag induced good (but reduced Tat) immune responses to all three antigens, which controlled virus load and protection from disease progression, even after very high dose challenge, beyond 50 weeks.

These results suggest that vaccine strategies based on a combination of immune targets distributed over both regulatory and structural viral antigens, capable of eliciting a diverse array of immune effector mechanisms will be most effective in controlling virus replication [24].

In a second experiment, each of four monkeys received a repeated primary IM/ID DNA immunization followed by two injections with recombinant Modified Vaccinia Ankara (MVA) expressing the corresponding set of antigens. MVA booster immunizations were given either IM/ID (group D) or intranasally (group E). Controls were naïve monkeys (group A), or animals that received the identical dose of empty vectors administered via the same routes (group B, C). All rhesus macaques were challenged with 40 MID50 of $SHIV_{89.6P}$ via the rectal route. All immunized animals revealed a 1-2 log virus load reduction in initial viraemia and at set point. Three out of four intranasally boostered animals (group E) and one of the animals with exclusively i.m./i.d. injections managed to fully eliminate the virus after 4 weeks as determined by quantitative PCR analysis. Stringent virus containment was paralleled by sustained levels of CD4 positive T cells. These data suggest that stringent virus containment in a relevant animal model can be provided depending on the type and route of delivered immunogens.

(iv)b Recombinant NYVAC

The NYVAC (vP866) strain was derived from a plaque-purified isolate (VC-2) of the Copenhagen vaccine strain whose genomic sequence is known by the precise deletion of 18 open reading frames (ORFs) from the viral genome. Among the ORFs deleted are two genes involved in nucleotide metabolism - the thymidine kinase and the large subunit of the ribonucleotide reductase; the gene encoding the viral hemagglutinin; the remnant of a highly expressed gene responsible for the formation of A-type inclusion; the disrupted gene normally encoding a serine protease inhibitor; and a block of 12 ORFs bounded by two known viral host range regulatory functions. Within this block, a secretory protein (ORF N1L) implicated in viral virulence and a functional complement 4b binding protein are encoded. The ORFs were deleted in a manner which prevents the synthesis of undesirable novel gene products. The net result of these deletions is a highly attenuated vaccinia virus which nonetheless retains the capacity to evoke strong immune responses to foreign immunogens.

The NYVAC strain was demonstrated to be highly attenuated, to retain a broad host range tropism and to be safe in several animal toxicology studies. More specifically, inoculation of NYVAC (5×10^8 pfu) caused no lesion or apparent disease in nude mice and did not replicate to any appreciable degree in immunocompromised SIV-infected macaques when administered at 10^8 pfu by the i.m, s.c or i.v route.

NYVAC-HIV C clade (vP2010)
The multigenic recombinant NYVAC vP2010 (env, gag, pol, nef of HIV CRF_07) has been shown to be stable for the HIV genes.

Immunogenicity and challenge data in animal models
Mice and macaques inoculated with NYVAC-SIV, NYVAC-HIV-1 or NYVAC-HIV2 clade B recombinants have elicited SIV, HIV1 or HIV2-specific neutralising antibodies. Non-human primates vaccinated with NYVAC-SIV*env/gag-pol* have been “protected” against an i.v. or i.r. challenge with the highly pathogenic SIV_{mac251} strain. Macaques primed with NYVAC-HIV1*env* or NYVAC-HIV1*env/gag-pol* and boosted with HIV-1 gp120 or peptide have been protected against HIV2 challenge and macaques primed with NYVAC-HIV2*env/gag-pol* or NYVAC-HIV2*env* and boosted with an HIV-2 envelope have been “protected” against an i.v. HIV-2 challenge. These studies indicated that a significant percentage of monkeys vaccinated with NYVAC-SIV, NYVAC-HIV1 or NYVAC-HIV2 recombinants can clear a pathogenic SIV or HIV-2 challenge or control SIV or HIV-2 infection following a viral challenge. Three NYVAC-based candidate human vaccines against rabies (NYVAC-RG containing the glycoprotein G gene), Japanese encephalitis virus (NYVAC-JEV expressing four JEV antigens) [25] and malaria (NYVAC-Pf725 harboring 7 plasmodium genes) [16] have been evaluated in Phase 1/2 trials in France or the United States. They have been found to be safe and well tolerated, although local reactions tend to be more marked than those seen with licensed recombinant immunogens. There is also evidence that these NYVACs induce cellular immunity.

(v) Rationale for this study
This study is preceded by two others: EV01 and EV02. In EV01 the safety and immunogenicity of NYVAC-C x 2 was assessed. EV02, which finished in 2006, provided additional safety data to EV01 on NYVAC-C, and the first immunogenicity data in humans on the use of DNA-C as a priming agent, comparing DNA-C x2 + NYVAC-C x2 to NYVAC-C x2 alone. The highest practicable dose of DNA-C was investigated in EV02 and a significantly higher proportion of volunteers that completed the prime-boost regimen had IFNγ ELISpot responses compared to those that received both NYVACs, 90% compared to 40% (2-sided Fisher's exact Test: $p = 0.003$).

During the implementation of EV02 a consensus began to emerge that three DNA immunisations may be required for optimal priming. Data from the exploratory DNA x3 prime – Ad5 x1 boost trial supporting this hypothesis were presented at the AIDS Vaccine Conference 2006 in Amsterdam, with higher mean responses and an increase in the breadth of response with most participants responding to gag as well as *env* [26].

This study proposes to compare the DNA-C x3 prime + NYVAC-C x1 boost to the DNA-C x2 prime + NYVAC-C x2 boost in a large enough study to make a robust assessment.

The design will be open to the participants and investigators, but the laboratory personnel conducting the ELISPOT, flow cytometry and antibody assessments will be blind to allocation. The randomisation will be stratified by gender to ensure an equal proportion of women in each group.

EuroVacc 03/ANRS Vacc 20 will address the following questions:
1. Does DNA-C x 3+NYVAC-C x1 elicit broader responses to peptide pools in IFNγ ELISpot assays compared to DNA-C x 2+NYVAC-C x2?
2. Does DNA-C x 3+NYVAC-C x1 elicit higher mean responses across the peptide pools in IFNγ ELISpot assays compared to DNA-C x 2+NYVAC-C x2?
3. Is DNA-C x 3+NYVAC-C x1 safe compared to DNA-C x 2+NYVAC-C x2?

II Summary of Trial

140 healthy volunteers at low risk of HIV infection will be entered into the study to receive one of the following regimens:

	Wk 0	Wk 4	Wk 8	Wk 20	Wk 24
group 1<br>"DNAx3"<br><br>n=70	DNA-C<br>2x2ml IM right and left vastus lateralis	DNA-C<br>2x2ml IM right and left vastus lateralis	DNA-C<br>2x2ml IM right and left vastus lateralis		NYVAC-C<br>1 ml IM<br>Non-dominant deltoid
group 2<br>"DNAx2"<br><br>n=70	DNA-C<br>2x2ml IM right and left vastus lateralis	DNA-C<br>2x2ml IM right and left vastus lateralis		NYVAC-C<br>1 ml IM<br>non-dominant deltoid	NYVAC-C<br>1 ml IM<br>Non-dominant deltoid

Participants will attend the clinical centre on at least 14 occasions over 72 weeks. These visits will comprise:

- Screening
- Enrolment, trial entry and first immunisation
- Second immunisation
- Third immunisation
- Fourth immunisation
- Early follow-up after each immunisation
- Two other follow-up for safety and immune responses
- Week 48 final safety visit
- Week 72 immune responses

Procedures
Participants will have blood and urine collected, and receive the immunisations as outlined in the schedule (see 2.6). They will be counselled prior to and following a HIV test, and given health education on prevention of sexually transmitted infections including HIV.

Endpoints
The primary end-points are:

- Immunogenicity: cellular responses to *env, gag, pol* and *nef* assessed using the IFN-γ ELISPOT technique. The two treatment groups will be compared in terms of the proportion responding to *env* plus at least one of the *gag, pol, nef* peptide pools at weeks 26 or 28 using the Chi-square test.
- Safety: local (pain, cutaneous including induration), general (fever, chills, headache, nausea, vomiting, malaise, myalgia) and other unsolicited adverse events within 7 and 28 days.

Institutional responsibility in trial
Product supply and indemnity for product:

- The EuroVacc Foundation will be responsible for the provision of indemnity for any claim by a clinical trial participant in respect of a SAE attributable to participation in the clinical trial, other than clinical negligence in the EuroVacc centres.

- The ANRS will be responsible for the provision of indemnity for any claim by a clinical trial participant in respect of a SAE attributable to participation in the clinical trial, in the ANRS Centres.
- The ANRS will act as the legal representative of Eurovacc foundation in the EU member states.
- Each clinical centre will hold indemnity for clinical negligence

Coordination of the data management and monitoring adherence to ICH GCP will be shared between the UK Medical Research Council Clinical Trials Unit (MRC CTU) and the INSERM U593 CTU, with responsibility for the EuroVacc centres and the ANRS centres respectively. The enrolment and serious adverse event reporting numbers are:

EuroVacc centres - tel: 00 44 (0)207 670 4783 Fax: 00 44 (0)207 670 4814
ANRS centres - tel: 00 33 (0)557 57 1392 Fax: 00 33 (0)557 57 1172

An analysis plan will be developed by these two institutions in collaboration with all the participating investigators.

The EuroVacc trials are overseen by a Trial Coordinating Committee (TCC). The committee has an independent chair, Professor Geoffrey Schild, and two other independent members. The two Chief Investigators listed on this protocol will represent the EuroVacc/ANRS investigators and have voting rights on this committee. Additional non-voting members may attend as appropriate to the contents of the meeting. This committee will be responsible for final decisions about grade of adverse events and relationship to study vaccine, and will be determined by the evolution of the adverse event.

An entirely independent Data and Safety Monitoring Committee has been appointed to review the design and protocol, and will meet on one occasion during the trial, to review the safety data, after half the participants have completed the immunisation regimen and passed week 28. Using this dataset the investigators will review the pooled immunogenicity data blind to allocation and, if less than 60% of participants have ELISpot responses, then the DSMC will be asked to review the immunogenicity by allocation as well as the safety data, and make a recommendation based on the upper confidence bound of the difference between the two groups (DNAx3 – DNAx2). The members of this committee are:

Prof Tim Peto	Chair and infectious diseases specialist
Dr Ferdinand de Wit	Clinical Epidemiologist
Dr Jürg Schifferli	Clinical Immunologist
Dr Jean Pierre Aboulker	Biostatistician

There will be an additional meeting if 3 or more participants experience an unexplained, unexpected grade 3 or 4 clinical or laboratory event (confirmed on attendance or repeat testing) not resolved within 72 hours and considered probably or possibly related to vaccine product.

Screening will not commence until the approvals appropriate to the clinical centre according to ICH GCP have been obtained.

III Scientific questions to be addressed:

Regimen	Wk 0	Wk 4	Wk 8	Wk 20	Wk 24
group 1 ”DNAx3” N=70	DNA C 2x2ml IM right and left vastus lateralis	DNA C 2x2ml IM right and left vastus lateralis	DNA C 2x2ml IM right and left vastus lateralis	Nothing	NYVAC C 1 ml IM Non-dominant deltoid
group 2 ”DNAx2” N=70	DNA C 2x2ml IM right and left vastus lateralis	DNA C 2x2ml IM right and left vastus lateralis	Nothing	NYVAC C 1 ml IM Non-dominant deltoid	NYVAC C 1 ml IM Non-dominant deltoid

**Primary Questions**	**Datasets**	**Primary safety and immunogenicity end-points**
Is there a ~~quantitative~~ difference in the breadth of immune responses following the 3 prime regimen compared to the 2 prime regimen?	Wks 26, 28 IFNγ ELISPOT<br>1 vs 2	The proportion of individuals in each group with CD8/CD4+ T cell responses to env plus at least one of the gag, pol, nef peptide pools
**Secondary Questions**		
Is there a quantitative difference in the strength of immune responses following the 3 prime regimen compared to the 2 prime regimen?	Wks 26, 28 IFNγ ELISPOT<br>1 vs 2	Mean IFNγ ELISPOT response across peptide pools and wks 26 and 28 per group
Is there a quantitative and/or qualitative difference in CD4 and CD8 T-cell responses following the 3 prime regimen compared to the 2 prime regimen?	Wks 26, 28<br>Flow cytometry : Intracellular synthesis of IL-2/ IFNγ<br>1 vs 2	Proportion of individuals in each group with CD4 and CD8 T cell responses to HIV peptide pools assessed by the percentage of cells producing IL-2 and/ IFNγ. Comparisons between groups of mean percentages of HIV specific CD4 and CD8 T cells.
Is the 3 prime regimen safe compared to the 2 prime regimen?	All weeks<br>1 vs 2	Proportion of participants per group with grade 3 or above adverse events during the trial, excluding those considered unrelated to vaccine

Detailed study schedule in section 2.6

1.0 General plan

1.1 Objectives

The primary objectives are to compare the immunogenicity and safety of the three DNA-C prime and one NYVAC-C boost regimen to two DNA-C prime and two NYVAC-C boosts in healthy volunteers at low risk of HIV infection.

1.2 Design and duration of the study

1.2.1 Design

This is a randomised phase I/II international trial with a parallel group design, open to participants and investigators but blind to laboratory personnel.

1.2.2 Start of enrolment and duration of the study

The plan is to start the study in Q1 2007, with the aim to enrol within 9 months. The duration of the study per participant is 72 weeks. The duration of participation from screening to final visit is a maximum of 78 weeks per participant.

1.3 Population

The study will require 140 healthy male and female volunteers who are HIV negative and at low risk of HIV infection recruited through several clinical centres.

Inclusion Criteria

- age between 18 and 55 years on the day of screening
- available for follow-up for the duration of the study (78 weeks from screening)
- able to give written informed consent
- at low risk of HIV and willing to remain so for the duration of the study
  low risk of HIV infection defined as:
  - no history of injecting drug use in the previous ten years
  - no gonorrhoea or syphilis in the last six months
  - no high risk partner (e.g. injecting drug use, HIV positive partner) either currently or within the past six months
  - no unprotected anal intercourse in the last six months, outside a relationship with a regular partner known to be HIV negative
  - no unprotected vaginal intercourse in the last six months outside a relationship with a regular known/presumed HIV negative partner
- willing to undergo a HIV test
- willing to undergo a genital infection screen
- if heterosexually active female, using an effective method of contraception with partner (combined oral contraceptive pill; injectable contraceptive; IUCD; consistent record with condoms if using these; physiological or anatomical sterility in self or partner) from 14 days prior to the first vaccination until 4 months after the last, and willing to undergo urine pregnancy tests prior to each vaccination
- if heterosexually active male, using an effective method of contraception with their partner from the first day of vaccination until 4 months after the last vaccination
- for French volunteers only :
  - subjects registered in French Health ministry computerised file and authorised to participate in a clinical trial
  - subjects covered by Health Insurance
  - subjects included in the ANRS vaccine research network of volunteers

Exclusion Criteria

- pregnant or lactating
- clinically relevant abnormality on history or examination including history of grand-mal epilepsy; severe eczema; allergy to eggs or gentamicin; severe allergic diseases; liver disease with inadequate hepatic function; haematological, metabolic or gastrointestinal

disorders; uncontrolled infection; autoimmune disease, immunodeficiency or use of immunosuppressives in preceding 3 months
- receipt of live attenuated vaccine within 60 days or other vaccine within 14 days of enrolment
- receipt of blood products or immunoglobin within 4 months of screening
- participation in another trial of a medicinal product, completed less than 30 days prior to enrolment
- history of severe local or general reaction to vaccination defined as

  local: extensive, indurated redness and swelling involving most of the antero-lateral thigh or the major circumference of the arm, not resolving within 72 hours

  general: fever >= 39.5°C within 48 hours; anaphylaxis; bronchospasm; laryngeal oedema; collapse; convulsions or encephalopathy within 72 hours
- HIV 1/2 positive or indeterminate on screening
- positive for hepatitis B surface antigen, hepatitis C antibody or serology indicating active syphilis requiring treatment
- positive for DNA/ANA antibodies at titre considered clinically relevant by immunology laboratory
- grade 1 or above routine laboratory parameters (see section 4.1.4 & appendix 4 for definitions).

  Note of clarification 18th April 2008: hyperbilirubinemia has to be considered as an exclusion criterion only when confirmed to be conjugated bilirubinemia.
- unlikely to comply with protocol

1.4 Trial products

1.4.1 Supply, storage and composition of NYVAC C

Transgene will be responsible for manufacture and supply of this clinical material according to Good Manufacturing Practice. The product is presented as a colourless solution at 1 mL per ampoule in single-use 2 mL glass ampoules and should be stored at –20°C or below. The composition is given below:

Name of Ingredient	Concentration	Content/Ampoule
NYVAC-HIV (vP2010)	≥ 1.0×10^7 pfu / ml	≥ 1.0 ml
Other Ingredients		
Tris Buffer	1.21 g/L	0.25 mL
Lactoglutamate (Virus stabilizer)		0.25 mL
Freezing medium	According to formula 65-1-3	0.50 mL

1.4.2 Preparations prior to use of NYVAC C

Prior to use the ampoules will be thawed at room temperature for up to one hour. When completely thawed the ampoules should be gently swirled. Care must be taken not to shake or invert the ampoules.

1.4.3 Supply, storage and composition of DNA C

Cobra will be responsible for bulk manufacture of this clinical material, release testing and technical release of vialled product, and ProPharma for the preparation of the vialled material, including labelling. All procedures will be according to Good Manufacturing Practice. The presentation is in liquid form, with an extractable volume of 2ml to 2.2ml in 5ml vials which should be stored at –20°C or below. The appearance is clear and the composition is given below:

**Name of ingredient**	**Quantity per ml of DNA HIV-C vaccine**
DNA	1.0 mg
Tris-HCl	1.57 mg
EDTA	0.372 mg
NaCl	9mg
Water for injections	To 1 ml

1.4.4 Preparations prior to use of DNA C

Prior to use the vials will be thawed at room temperature. When completely thawed the vials should be gently swirled. Care must be taken not to shake or invert the vials.

1.4.5 Labels

NYVAC C and DNA C will be packaged by Transgene and Cobra respectively. The trial products will be in ampoules or vials pre-labelled according to standardised operating procedures and statutory regulatory requirements.

Cartons will be supplied to the pharmacist responsible for each site for storing the used ampoules and vials labelled according to standardised operating procedures and statutory regulatory requirements.

1.4.6 Dispensing records and disposal of unused product

The designated pharmacist will, upon receipt of supplies prior to commencement of the trial, conduct an inventory and complete a receipt, one copy of which will be retained at the site, one copy forwarded to MRC CTU (EuroVacc centres) or the INSERM U593 CTU (ANRS centres) and the original returned to the supplier. During the trial the pharmacist will be responsible for reviewing the dispensing log.

On the day of immunisation, and with the participant present, the Investigator will complete a prescription with the trial number, date of birth, and immunisation number (DNA 1, 2 or 3, NYVAC 1 or 2). The immunisation number and date will be entered against the trial number in the dispensing log. The ampoule/vial label will be cross-checked against the details on the prescription and dispensing log by two individuals, prior to product being administered.

The individual who administers the injection will be responsible for ensuring that the return of the used ampoules/vials is recorded in the dispensing log at the end of the clinical session, and that they are placed in the appropriate participant carton.

At the end of the trial all used and unused ampoules/vials will be checked against the inventory by staff from MRC CTU/INSERM U593 CTU before return to the supplier or disposal on site according to local pharmacy guidelines and applicable regulations. Documentation of disposal will be provided to the MRC CTU/INSERM U593 CTU, the Foundation and both suppliers.

During the trial, product accountability will be monitored by the prescriptions, the dispensing log, the returns, the trial register and data collected on the case report forms.

1.5 End-points

1.5.1 Primary

The primary endpoints are immunogenicity and safety as defined below.

The primary immunogenicity parameter will be the presence of CD8/CD4+ T cell responses defined according to internationally agreed criteria for evaluation of IFNγ ELISPOT assays (see section 8),

- in response to *env* *plus* *at least one of the gag, pol, nef peptide pools*
- at weeks 26 or 28

The primary safety parameters will be graded according to appendix 4, and are:

- Grade 3 or above local adverse event (pain, cutaneous reactions including induration)
- Grade 3 or above systemic adverse event (temperature, chills, headache, nausea, vomiting, malaise, and myalgia)
- Grade 3 or above other clinical or laboratory adverse event confirmed at examination or on repeat testing respectively.
- Any event attributable to vaccine leading to discontinuation of the immunisation regimen.

Note of clarification 8th Nov 2006: This will capture all Serious Adverse Events (SAE) as defined by ICH GCP as well as other grade 3 and 4 events that do not meet the criteria of an ICH GCP SAE.

Data on local and systemic events listed above will be solicited with specific questions or using a diary card for a minimum of 7 days following each immunisation. Data on other clinical events and laboratory events will be collected with an open question at each visit and through routine scheduled investigations respectively.

1.5.2 Secondary

Secondary immunogenicity and safety end-point information will be collected on all participants on the following:

- cellular responses
  - CD8/CD4+ T cell mean IFNγ Spot Forming Units (SFU) per million cells across the peptide pools at weeks 26 and 28
  - CD8/CD4+ T cell mean Spot Forming Units (SFU) per million cells across the peptide pools at any week following the first immunisation including weeks 48 and 72
  - mean proportion of CD4/CD8+ T cells producing IL-2 and/or IFN-γ following ex-vivo stimulation with HIV-1 peptide pools at weeks 26 and 28, 48 and 72
  - number of different epitopes that can be characterised
- antibody responses
  - precise assays to be determined at a later stage, but prior to unblinding of laboratory personnel
- all grade 1 and 2 adverse events
- all events including those considered unrelated

2 Schedule of Visits

2.1 Recruitment

Healthy volunteers will be recruited through the ANRS network of volunteers or through advertising in hospitals, colleges, newspapers and magazines and given a telephone number to contact. They will be provided with further information about the study, and asked to complete a short interview (by telephone or in person) to assess their suitability. At this point they will be allocated a number from the screening register. They will be given or sent an

information sheet (appendix 1) about the trial. The information to be provided and collected will be defined in the trial specific operating procedures and proformas.

If they are still interested and willing to participate, they will be invited to attend for screening.

2.2 Screening

At this visit, which must take place within 42 days of the enrolment visit, the trial will be discussed in detail, and a more detailed check of eligibility conducted using a case record form to standardise this procedure. Any questions about the study will be answered. If volunteers are still willing and interested they will be asked to sign part 1 of the informed consent form (appendix 2).

To ensure informed consent, subjects will go through the following processes in detail with a member of the study team

1) Pre-HIV test counselling
2) Safe sex counselling
3) That it is unknown whether or not the study vaccines will protect against HIV infection
4) That following immunisation they may develop antibodies that will produce a positive reaction in a routine HIV test, but that provisions have been made to distinguish between a post vaccination response and HIV infection during and after the trial
5) The level of care that will be made available to them should they be found to be HIV infected at any time during their participation in the study, including the screening period.
6) That they, or their partner should continue to use a reliable form of contraception for 14 days prior to the immunisation period and for 4 months afterwards
7) That they should continue to use condoms with sexual partners whose HIV status is not known
8) That they may be subject to social risk if they develop HIV antibodies, or by revealing their participation in the study

After informed consent has been collected, assessments and procedures will be undertaken according to the schedule 2.6, including a physical examination and collection of specimens for laboratory investigations. Details of these are given in sections 3 and 4. The investigator will enter the data collected onto the case record form and on completion this will be sent promptly to the MRC CTU/INSERM for entry onto the trial database.

French volunteers should be registered in the French Health ministry computerised file by the investigators with a suspension duration of four months from the end of the study.

2.3 Enrolment and Trial Entry

An enrolment list will be prepared by the CTU.

The results of the screening investigations will be reviewed and volunteers who are still eligible and willing will be asked to complete part 2 of the informed consent. This will include asking for consent to undertake long-term follow-up by direct contact. They will be strongly advised to inform their current general practitioner of their participation but this will not be a pre-requisite to enrolment. Assessments and procedures will be undertaken according to the schedules in 2.6, and data entered on the case record form.

Participants will be enrolled by completing the appropriate case report form and contacting the MRC CTU (EuroVacc centres) or INSERM U593 CTU (ANRS centres), with the participant present. Eligibility will be checked, the trial number confirmed and regimen allocated.

The enrolment number is:
EuroVacc centres - tel: 00 44 (0)207 670 4783 Fax: 00 44 (0)207 670 4814
ANRS centres - tel: 00 33 (0)557 57 1392 Fax: 00 33 (0)557 57 1172

Trial product will be dispensed as outlined in 1.4.3.

2.3.1 Immunisations

Weeks 0, 4 and 8

Two 2ml immunisations will be given at 0, 4 and to half the participants at 8 weeks, one into each vastus lateralis muscle in order to deliver 4ml total of the DNA C at each timepoint according to published guidelines [27].

Weeks 20 and 24

At weeks 20 and 24 or week 24 alone, a single immunisation will be administered into the deltoid muscle of the non-dominant arm.

During the procedure, regardless of muscle, the overlying skin will be stretched flat prior to insertion of the needle. To ensure that the needle reaches the muscle, and that product does not seep into the surrounding subcutaneous tissues, a decision about the needle length required will be made individually for each participant, based on published recommendations [27,28]. Ten minutes after immunisation, the site will be inspected and any local reactions recorded on the case record form. Following the NYVAC immunisation inspection, a plaster will be placed over the site.

The immunisations will occur in an outpatient setting, and participants will be closely observed for one hour after each immunisation, at which point vital signs (pulse, blood pressure and respiratory rate) will be recorded on the case record form, as will any local or systemic reaction.

The plasters, needles and syringes used for all immunisations will be autoclaved or placed in hypochlorite solution at the end of the clinical session.

2.3.2 Follow-up in the days following immunisation

A diary card will be given, with instructions and a full verbal explanation, for participants to record local and systemic adverse events for at least 7 days following immunisation, as well as medication taken.

Participants will be observed on the day of immunisation as above. Contact will be maintained by the clinical team, using a method chosen by the participant, as indicated by the evolution of adverse events, up to resolution of solicited local and systemic events. Additional visits may be recommended at the discretion of the clinical and principal investigators, if clinically indicated or in order to clarify observations.

2.4 Follow-up visits

Assessments and procedures will be performed according to schedules in 2.6.

2.5 Final visit

Assessments will be undertaken according to schedules in 2.6.

2.5.1 Reimbursement

Participants will be reimbursed for their travel expenses and any inconvenience caused according to site specific procedures, and this will be made clear to them in the information sheet. During the study participants will receive latex condoms free of charge. If any medication is required as a result of the study, this will also be provided free of charge.

2.5.2 In the event of discontinuation

It is possible that a participant may develop a condition, which regardless of the relationship to study product, may cause the Investigator to discontinue them from further immunisations. Follow-up should carry on whenever possible up to the time of the final visit, but at least until resolution or stabilisation of the condition. The date that the participant is discontinued from further immunisations and the reason will be recorded in the case record form.

2.6 EV03 Study schedule

Visit Number	1	2	3	4	5	6	7	8	9	10	11	12	13	14	15
Time in wk (+/-wk or d)	≤-6	0	1 (+/-1d)	4 (+/-3d)	5 (+/-3d)	8 (+/-3d)	9 (+/-3d)	12 (+/-3d)	20 (+/-1w)	22 (+/-1w)	24 (+/-1w)	26 (+/-1w)	28 (+/-1w)	48(+/-2w)	72 (+/-3w)
Immunisation group 1		X		X		X				No visit	X				
Immunisation group 2		X		X			No visit		X		X				
Eligibility	X	X													
History & exam[a]	X	X												X	
STI screen[b,c]	X														
HIV risk screen	X								X						
HIV Ab test[c]	X								(X)				X		
Safe sex counselling	X														
Adverse event assessment		X	X	X	X	X	X	X	X	X	X	X	X	X	
Haematology	X		X	(X)	X	(X)	X	(X)	X	X	(X)	X		X	
Chem pathology	X		X	(X)	X	(X)	X	(X)	X	X	(X)	X		X	
CD4 no & % & immnuoglob[ns]	X												X[f]		
DNA/ANA antibodies	X							X					X		
urine } Urinalysis	X		X	(X)	X	(X)	X		X	X	(X)	X	X	X	
urine } Pregnancy test, if female[c]		X		X		Group 1			Group 2		X			X	
Cell storage for HLA[d]				X											
Sera storage[d]		X				X		Group 1	Group 2			X	X	X	
ELISPOT responses		X		X		X		Group 1	Group 2		X	X	X	X	X
Flow cytometry		X										X	X	X	X
Cell storage		X		X		X		Group 1	Group 2		X	X	X	X	X
Diary card[e]		↔		↔			↔			↔	↔				

[a]including weight in kg, height in cm and arm & thigh circumference in cm
[b]including serology for syphilis, hepatitis B and C at screening. Genital swabs or urine for Neisseria gonorrhoea, chlamydia trachomatis and trichomonas vaginalis will be collected if indicated according to clinical standardised operating procedures (SOPs).
[c] additional HIV/STI and pregnancy tests will be performed if indicated by a change in risk status or menstrual history respectively
[d]HLA genotyping will be performed for evaluation of epitopic responses in responders; serological assays to be determined at a later stage, but prior to unblinding of laboratory personnel
[e]diary card will be completed for at least 1 week following immunisation
[f]CD4 number and % only at this visit
(x) means that a specimen will be collected only if indicated on history

3 Procedures

3.1 Blood and urine collection

Blood will be collected using a sterile needle, usually from the ante-cubital fossa, according to the schedule and transported to the appropriate laboratories. Urine will be collected into a sterile container as indicated on the schedule and either transported to the appropriate laboratory or tested by a member of the clinical team according to trial specific standardised operating procedures.

3.2 Clinical history and examination

A past medical history will be collected using the screening proforma at the screening visit, including details of any previous reaction to vaccination, history of epileptic fit, exposure to vaccinia, and contraceptive practices. The general examination will include weight (kg), height (cm) and arm and thigh circumference (see 4.1.1), blood pressure, inspection of the skin to exclude severe eczema and check for the presence of a vaccinia scar, respiratory, cardio-vascular and abdominal systems examination. An assessment of cervical and axillary lymph nodes will also be undertaken. The examination results and history of exposure to vaccinia will be recorded on the case record form. The general examination will be repeated at the final study visit.

3.3 Genital infection screen

The following will be collected in all participants

- serology for syphilis
- serology for markers of hepatitis B surface antigen or hepatitis C antibody

The following will be collected if indicated on account of risk and symptoms, according to trial specific clinical standardised operating procedures:

- urethral, vaginal, cervical and rectal specimens as appropriate for Neisseria gonorrhoea
- collection of urine for Chlamydia trachomatis
- vaginal swab for Trichomonas vaginalis

3.4 Pre-HIV test screen and counselling

Study personnel will assess volunteers for past and current risk of HIV infection using the screening proforma and counsel them prior to collecting blood for a HIV test. The counselling process will ensure that volunteers have sufficient knowledge about HIV infection to understand what the test is for, the implications of a positive, negative and equivocal result and the standard of care available for HIV infection locally. They will also be informed how and when they will receive the result, according to the local policy in each clinical centre.

3.5 Safe sex counselling and condom provision

Participants will be counselled by study personnel about the importance of condoms at screening and reminded on the day of each immunisation, or in the case of the third immunisation, at the preceding visit 2 weeks earlier. Hypo-allergenic condoms will be provided free of charge to participants throughout the trial.

3.6 HIV related issues

Please see section 6 for details of the procedures which may be required in the event of:

1) a request for a HIV test
2) HIV infection
3) Social discrimination as a result of post-vaccine response

3.7 Discontinuation procedures (including withdrawal)

Participants may withdraw at any time if they wish to do so, for any reason. The date of withdrawal and reason for doing so should be recorded in the appropriate case record form.
The clinical investigator may decide that it is not in the best interests of the participant to proceed to the next immunisation following an adverse event (see section 5.4.1 for recommendations). The discontinuation and reason should be recorded in the appropriate immunisation and reportable adverse event case record forms, which should be forwarded to

MRC CTU within the next 2 working days. Trial visits should carry on at least until resolution or stabilisation of the event, but ideally up to the last visit in the schedule, provided the participant is willing. The frequency of visits and laboratory investigations may be reduced on consultation with the Principal Investigator or Trial Management Group (see section 7).

3.8 Unblinding procedure

This is an open trial for clinical investigators and participants.

4 Assessments

4.1 Safety assessments

4.1.1 Local adverse events

Pain in the muscle injected will be graded by the participant according to the criteria in appendix 4 as mild (1) moderate (2), severe (3) or extreme (4) and recorded in the appropriate case record form or once the participant has left clinic, in the diary card.

Redness will be recorded as the maximum diameter and expressed as a proportion of the arm or thigh circumference and graded on this criteria and the presence of symptoms according to appendix 4. Arm circumference will be measured at screening at the point one third of the way down from the acromio-clavicular joint towards the elbow joint of the non-dominant arm, and thigh circumference half way between the iliac promontory and the patella. Participants will be asked to record the maximum diameter in the diary card, as well as the presence of any itching or other discomfort and any medication taken for relief of symptoms.

Blistering (vesiculation) or ulceration will be graded according to size, depth, time to healing and character of blisters (blood-filled).

The presence of induration (hardened swelling) following intramuscular injection will be considered grade 3 if considered by the clinician to have arisen from a pathological process within the muscle. Induration considered in the judgement of the clinician to result from product leaking into the intradermal or subcutaneous layers will be graded according to the intradermal parameters in the toxicity table (Appendix 4). Soft swelling local to the injection site is not considered induration and should be graded according to the soft swelling parameters in the toxicity table (Appendix 4).

Clinical staff will complete the case record form following each immunisation. Completed case record forms and the diary cards will be sent to the data coordination centres to be recorded on the database. In the event that there are two observations on the same day, those confirmed at a visit will take precedence over diary card observations in the analysis.

4.1.2 Systemic adverse events

Temperature will be measured by the oral route prior to immunisation and one hour later by study personnel, and graded according to appendix 4. Participants will be given digital thermometers to record their temperature in the diary card on the evening of immunisation, and daily thereafter for 7 days, and if still raised, they will be advised to continue to monitor their temperature daily until it returns to normal. The temperature observed by the clinical team will be recorded on the case record forms for visits.

Chills, headache, nausea, vomiting, malaise and myalgia will be graded by the participants according to appendix 4, recorded in the diary card on the evening following immunisation and daily for 7 days, or until resolution of symptoms whichever is longer. On days where a visit coincides with the diary card, the clinical staff will collect the information directly and record it on the case record form, and this will take precedence over diary card entries in the analysis.

4.1.3 Other adverse events

These will be recorded as reported following an open question to participants, with the

dates of commencement and resolution, and any medication required. They will be graded according to the general principles outlined in appendix 4. Social harm will also be recorded as an adverse event, graded according to the general guidelines.

4.1.4 Routine laboratory and urinary parameters

The following safety assessments will be undertaken in laboratories local to the clinical centre according to standard procedures subject to quality control:

- haematology: haemoglobin, white cell and platelet count, neutrophils and lymphocytes
- chemical pathology: liver function tests (AST/ALT, alkaline phosphatase, bilirubin), creatinine, glucose

<u>Note of clarification 18th April 2008</u>: conjugated bilirubin will take preference over total bilirubin for the purposes of grading hyperbilirubinemia

- immunology CD4 number and percentage; immunoglobulins A, G and M; antibodies to DNA and ANA

The following assessment will be undertaken on a specimen of urine, conducted by a member of the study staff according to trial specific standardised operating procedures:

- urinalysis: dipstick conducted by clinical staff and recorded normal/abnormal

If abnormal a mid-stream urine specimen will be collected in order to grade the event according to appendix 4.

4.2 Immunogenicity assessments

4.2.1 Sera storage

Sera samples will be collected and stored for evaluation of antibody responses that will be decided at a later stage, but prior to unblinding of laboratory personnel.

4.2.2 Cellular responses

Primary cellular responses will be assessed using the ELISPot assay on frozen specimens according to standardised operating procedures (SOPs) in a single accredited laboratory (CHUV), subject to ongoing quality control. The ELISPot reaction per well will be measured in an automated system, according to operating procedures that define the validity of the assay. All specimens will be sent to CHUV for the primary analysis.

CD4 and CD8 T cell responses will be assessed by flow cytometry on frozen specimens according to standardised procedures in a centralised INSERM laboratory at Créteil (CHU Henri Mondor). All specimens will be sent to CHU Henri Mondor for flow cytometry analyses. T-cell responses will be evaluated by the percentages of cells producing IL-2 and/or IFN-γ within CD4 and CD8 T cell populations after 18 hours of ex-vivo stimulation of PBMCs with HIV-1 peptide pools. HIV-1 clade C peptide pools will be provided by Eurovacc. Flow cytometry analyses will be performed at weeks 26, 28, 48 and 72.

4.2.3 Store for HLA testing

A store will be collected at week 4 to perform HLA typing in case this is required to assist future evaluation of the cellular responses.

4.3 Other assessments

4.3.1 HIV antibody test

Samples will be tested in the laboratories local to the clinical centre using ELISA, according to standard procedures subject to quality control.

4.3.2 Pregnancy test

A pregnancy test will be performed by analysis of a urine sample for Human Chorionic Gonadotrophin (HCG) collected from female participants at screening and on the day of each immunisation. The analysis will be conducted by a member of the study team according to trial specific standardised operating procedures.

4.3.3 Genital infection

The assays will be conducted in laboratories local to the clinical centre, according to standard procedures subject to quality control.

4.3.4 Following adverse event

Other assessments may be performed as clinically indicated due to an adverse event.

4.3.5 Concomitant medication

Participants will be asked about medication taken at each visit up to and including week 48, and this will be recorded in the case record form. It is expected that the name of the drug, indication for use, dose, frequency, start and stop dates will be available for prescription-only medication, either from the participant or from the prescribing physician. For medication available over the counter, the maximum information available on questioning the participant will be recorded.

5 Adverse events

5.1 Definitions

An adverse event is any adverse experience occurring during the course of the study from screening visit.

Criteria for grading clinical and laboratory events are listed in appendix 4.

A severe adverse event is one **graded 3 or 4** by criteria in appendix 4. Some, but not all grade 3 and 4 adverse events will be "serious" by ICH GCP criteria below.

5.1.1 Serious Adverse Events (SAEs)

An adverse event is considered to be a "serious adverse event" by ICH Good Clinical Practice (ICH GCP) criteria if it results in the following:

- death,
- a threat to life,
- requires in-patient hospitalisation or prolongs existing hospitalisation (hospitalisation for elective treatment of a pre-existing condition is not included),
- results in persistent or significant disability or incapacity,
- is a congenital anomaly (ie, the outcome of pregnancy involving a participant), or
- is any other important medical condition*.

*Examples of conditions regarded as "any other important medical condition" include allergic bronchospasm requiring intensive emergency treatment, seizures or blood dyscrasias which did not result in hospitalisation or development of drug dependency.

5.2 Relationship to study product

This can be classified as:

**Unrelated**	adverse events that can be clearly explained by extraneous causes and for which there is no plausible association with study product, or adverse events for which there is no temporal relationship
**Unlikely to be**	adverse events that may be temporally linked, but which are much more likely to be due to other causes than study product and which do not get worse with continuing use of product
**Possibly**	adverse events that could equally well be explained by study product or other causes, which are usually temporally linked and may improve when not using study product but do not reappear when using study product
**Probably**	adverse events that are temporally linked and for which the study

	product is more likely to be the explanation than other causes, which may improve when not using study product
**Definitely**	adverse events that are temporally linked and for which the study product is the most likely explanation, which disappear or decrease when not using study product and reappear when using study product

5.3 Reporting adverse events

Adverse events should be recorded on the appropriate case record form and reported to the MRC CTU (EuroVacc centres) or the INSERM U593 CTU (ANRS centres).

Any event resulting in discontinuation of the vaccination schedule should be reported within 2 working days of the decision to discontinue to the MRC CTU, INSERM U593 CTU, the Sponsors.

All SAEs should be reported to the MRC CTU, INSERM U593 CTU the same working day that the Clinical Investigator becomes aware of the event fulfilling the above criteria. This can be done by telephone or fax. The minimum criteria required in reporting a SAE are the participant identifiers (trial number/date of birth/initials), reporting source (name of Investigator), why the adverse event is identifiable as serious.

The adverse event reporting telephone number is
EuroVacc centres - tel: 00 44 207 670 4783/82 and fax: 00 44 207 670 4814
ANRS centres - tel: 00 33 (0)557 57 1392 Fax: 00 33 (0)557 57 1172

They will also be immediately reported to principal investigators by the CTUs.

Any SAE that is considered possibly, probably or definitely related to study product will be unexpected and as such qualify for expedited reporting to the regulatory authority.

Staff at the MRC CTU/ANRS Pharmacovigilance will confirm that the event qualifies in grade and relationship as a Suspected Unexpected Serious Adverse Drug Reaction (SUSAR) and arrange for multi-disciplinary review of the case to take place within 3 working days when the event is suspected to be possibly, probably or definitely related to study product. Those involved in this review will include the site Clinical Investigator, the medical expert at MRC CTU/ANRS pharmacovigilance and a panel of individuals with the expertise in quality and preclinical aspects of the vaccines and to the clinical responses to immunogens. The report will be prepared by the MRC CTU Medical Expert or their deputy (EuroVacc centres) or a physician from the ANRS Pharmacovigilance team (ANRS centres) and filed with the appropriate regulatory authorities, Ethics committees, and Eudravigilance, within the timelines required by national legislation. MRC CTU and INSERM U593 CTU will be responsible for sending a copy of the report to their other respective clinical centres involved in the programme and to inform the full Trial Coordinating and Data and Safety Monitoring Committees, the EuroVacc Foundation, Sanofi Pasteur and Cobra. For EuroVacc centres, the site Clinical Investigator is responsible for notifying their Local Research Ethics Committee. For ANRS centres; ANRS pharmacovigilance team will notify the Ethics Committee. The detailed process for the reporting and filing of SUSARs to the EMEA (Eudravigilance), the relevant regulatory authorities and the Ethics Committee, as well as the writing of the annual safety report will be further defined in a SOP.

Examples of SAEs that do not require expedited reporting include:

- Hospitalisation for scheduled surgery unrelated to vaccine, other than temporally (within 30 days)
- Orthopaedic or traumatic injuries requiring hospitalisation
- Hospitalisation planned for pre-existing conditions not due to an aggravation in the condition
- SAEs occurring more than 30 days after vaccination and that are expected not related to the vaccine or having appeared before vaccination without any

aggravation after vaccination

These must be prioritised for data entry and the site Principal Investigator is responsible for ensuring that the completed SAE case record form reaches data entry within 10 working days of becoming aware of the event.

5.4 Clinical management

Events will be managed by the clinical trial team who will assess and treat the event as appropriate, including referral to an independent physician and/or the participant's General Practitioner if required. There will be clinical operating procedures in place for the management of abnormalities detected following urinalysis or routine laboratory tests.

5.4.1 Recommendations for discontinuation

Discontinuation of the study vaccine schedule due to an adverse event is at the discretion of the site Principal Investigator or their Medical Deputy, but would be recommended in the event of a grade 3 or 4 clinical or laboratory event (confirmed on examination or repeat testing respectively) which did not resolve within 72 hours. There must be no further immunisations following:

1) extensive, indurated redness and swelling involving the major circumference of the arm or thigh, not resolving within 72 hours or
2) fever >= 39.5^{o}C within 48 hours; anaphylaxis; bronchospasm; laryngeal oedema; collapse; convulsions or encephalopathy within 72 hours

6 Management of HIV issues during and following the trial

6.1 HIV testing

Only volunteers with a negative HIV ELISA result will be enrolled. A risk assessment will be undertaken prior to the NYVAC-C immunisations and if risk status has changed then immunisation may have to be delayed until the HIV status of the participant is clarified. It is possible that participants may develop antibodies and test 'positive' in routine HIV ELISA assays subsequent to immunisation. Accredited laboratories local to each clinical centre will conduct any additional tests to distinguish between infection and a post-immunisation response required either for clinical management, or at the request of a participant. Entry and week 48 samples will be collected and tested in real-time so that participants can be informed of the result and a plan to recall and retest them made should this be necessary. In the event of ongoing post-immunisation positive ELISA, the participants will be invited to reattend annually until such time as this response has disappeared, and provided with an explanatory identity card in the interim.

6.1.1 Verification of HIV status of participants

If certification is required at the request of the participant, this can be provided by the clinical team after testing at the local laboratory.

Results will always be given by a member of the study team unless the participant requests an independent physician, in which case this will be arranged.

If a specimen from a participant suggests that they are HIV infected, a second specimen will be collected and retested.

6.2 HIV infection

In the unexpected circumstances that a participant in the trial acquires HIV infection, they will be managed in the following way:

6.2.1 Referral for clinical care

Participants will be referred initially to a specialist physician for a full discussion of the clinical aspects of HIV infection. Further investigations will be undertaken as necessary. Should the participant prefer to be managed at a hospital closer to their home, or by their General Practitioner, this will be arranged.

6.2.2 Referral for counselling:

This will be arranged by the specialist physician, to a counsellor at their clinical centre. The counselling process will assist the participant in the following issues:

- psychological and social implications of HIV infection
- who to inform and what to say
- implications for sexual partners
- avoidance of risk to others in future

6.2.3 Informing the General Practitioner

The participant will be encouraged to do this, but the decision will remain at the discretion of the individual.

6.2.4 Immunological follow-up

Follow-up of HIV infected individuals who have received study vaccine products will be determined by the Operational Sub-Group. The intensity of assessments will be dependent both on the number of immunisations received by the individual and their clinical progress, including changes in surrogate markers such as viral load and CD4 count.

6.3 Social discrimination as a result of a post-vaccine response

The aim is to minimise the possibility of social discrimination in participants who develop a positive HIV-ELISA test by providing HIV testing and certification for participants as required, outlined above, both during and after the trial. In addition, an identification card stating that the individual has participated in a vaccine trial, with a contact number in case of medical emergency, will be provided.

In the unlikely event that a participant suffers social discrimination as a result of a post-vaccination response, the clinical investigators will assist the participant.

7 Management of the trial

7.1 Data management at the Clinical Centre

Staff at the clinical centres will be responsible for:

- Entering relevant information (see section 7.5) in the clinical notes, and holding a record for each participant which includes the CRFs with any changes made signed and dated
- The accurate completion of the case record forms
- The prompt return of the completed forms to the MRC CTU/INSERM U593 CTU
- Notification of SAEs as soon as they become aware of the event to the MRC CTU/INSERM U593 CTU

Data will be recorded directly onto the CRFs, which will provide the majority of source data for the trial. There will be some additional source data in the clinical notes, such as medical history related to eligibility, dates of visits including immunisation, results of pregnancy tests, and details of clinical management (description of adverse events and concomitant medication).

CRFs will be supplied by the data management centres and these will be forwarded for data entry after completion according to standardised operating procedures. Copies of laboratory reports containing the results of routine haematology, chemical pathology and immunology may be sent instead of completing these sections of the CRF, provided they are clearly labelled with the trial number and the date of collection. A member of the clinical trial team must sign the laboratory report. In the event of an abnormality, an indication should be given whether or not action was taken, the date of review and the signature of the clinician reviewing the result.

Changes to the CRF should be signed and dated, including changes made before the form is returned to the data management centre.

CRFs and clinical notes should be kept in a secure location for 2 years after the last approval of a marketing application or until 2 years have elapsed since formal discontinuation of product development, and at least 10-15* years after the clinical trial has ended.

* as per local regulations

7.2 Data management in the immunology laboratories

Standardised operating procedures will be followed in all laboratories to ensure the quality of the data. Data will be stored electronically in an agreed format and datafiles transferred to the data management centres for the main analysis.

7.3 Data management at the MRC CTU & INSERM U593 CTU

MRC CTU/INSERM U593 CTU will share responsibility for:

- Design of the CRFs in collaboration with the Investigators
- The database applications that will contain the computerised trial data
- Data entry for the clinical trial other than the immunologic assays
- Monitoring the trial according to ICH GCP guidelines including monitoring vaccine accountability, and dispatch and arrival of immunological specimens
- Preparation of reports to assist the monitoring
- Holding a record for each participant which contains the copy of the CRF and documentation detailing all the changes made subsequent to monitoring visits, queries raised and how they were addressed
- Coordination of the committee and group meetings (section 7.6 and 7.7) in collaboration with the Investigators
- Development of the analysis plan and conducting the analyses
- Preparation of the analysis files from the database prior to analyses
- Coordination of the final report

All CRFs and laboratory reports returned to the data management centres will be reviewed by the Data Management Team, according to standardised operating procedures. CRFs will be checked for completeness and passed for review by the Medical Experts or their deputies if required. Data will be entered onto a computerised database. Consistency checks and range checks will be performed.

The accuracy of data entry will be checked according to standardised operating procedures at each data management centre.

The data manager or their deputy will review adverse events, as they arise. Queries raised will be directed to the investigators at the relevant clinical centre by letter, fax, email or at a monitoring visit.

Prior to analysis, the safety data will be checked, adverse events validated and data extracted in order for the trial statisticians to run the analysis and prepare the tables.

Once each CTU will have a complete and final set of data from their affiliated clinical centres, these two datasets will then be merged at INSERM U593 CTU.

7.4 Monitoring by MRC CTU & INSERM U593 CTU

Staff from the data management centres will visit the clinical centres they are responsible for to validate trial data held on the database against the clinical records. The site Principal Investigator and participants, by giving consent, agree that the MRC CTU/INSERM U593 CTU may consult and/or copy source records (clinical notes and laboratory values) in order to do this. Such information will be treated as strictly confidential and will in no circumstances be made publicly available. The monitoring will adhere to ICH Good Clinical Practice guidelines. The following data should be verifiable from source documents:

- documentation of any existing conditions or past conditions relevant to eligibility
- signed consent
- dates of visits including dates of immunisations

- a sample of reported laboratory results
- grade 3 or 4 adverse events and any events leading to discontinuation of the immunisation schedule
- concomitant prescribed medication

Vaccine returns will also be monitored at visits to the clinical site.

7.5 Data Ownership

The data generated in this study will be the property of the EuroVacc Foundation, the ANRS and the Investigators involved in the conduct of this trial and the combined database will be held on their behalf by one or both data management centres. If data are required at an earlier date for the purposes of regulatory submissions, a request should be made to the Trial Coordinating Committee.

7.6 Trial Coordinating Committee (TCC)

The supervision of the trial will be responsibility of the Trial Coordinating Committee (TCC). The committee has an independent chair, Professor Geoffrey Schild, and the voting members will include the two Chief Investigators involved in the conduct of this trial, and one further independent member. Representatives from EuroVacc and ANRS, from Imperial, Klinikum-UREG, ANRS centres and other additional non-voting members will attend as appropriate to the contents of the meeting. This committee will be responsible for final decisions about grade of adverse events and relationship to study vaccine. Notes of meetings will be kept, and these will be filed with the Sponsors.

The trial may be terminated by this Committee for any reason, including on the recommendation of the DSMC.

7.6.1 Operational sub-Group (OSG)

This group will oversee the day to day running of the trial and the members will be primarily the clinical and data management teams. The immunologists will join if there are relevant items on the agenda, but care will be taken to ensure they are not unblinded to allocation. Notes will be taken and will form the basis of the progress report to the Trial Coordinating Committee.

7.7 Data and Safety Monitoring Committee (DSMC)

An entirely independent Data and Safety Monitoring Committee has been appointed to review the design and protocol, and will meet on one occasion during the trial, to review the safety data, after half the participants have completed the immunisation regimen and passed week 28. Using this interim dataset the investigators will review the pooled immunogenicity data blind to allocation and, if less than 60% of participants have ELISpot responses, then the DSMC will be asked to review the immunogenicity by allocation as well as the safety data, and make a recommendation based on the upper confidence bound of the difference between the two groups (DNAx3 – DNAx2). The members of this committee are:

Prof Tim Peto	Chair and infectious diseases specialist
Dr Ferdinand de Wit	Clinical epidemiologist
Dr Jürg Schifferli	Clinician
Dr Jean Pierre Aboulker	Biostatistician

7.7.1 Indications for additional review

There will be an additional meeting if 3 or more participants experience an unexplained, unexpected grade 3 or 4 clinical or laboratory event (confirmed on attendance or repeat testing) not resolved within 72 hours and considered probably or possible and likely to be related to vaccine product.

7.7.2 Indications for discontinuation of immunisations in all participants

If 3 or more participant experience grade 3 or 4 adverse events attributed to the same product, then further recruitment to the Clinical Trial and immunisations with that product will be discontinued pending an unblinded review of all safety data by the

DSMC. Following this review the DSMC will make a recommendation to the Trial Coordinating Committee about continuation of the trial. The sponsors will formally decide to terminate or not the clinical Trial.

8 Statistical considerations

Analyses will be conducted according to a modified intention to treat principle, in which all randomised volunteers that received at least one immunisation will be included. Volunteers with IFNγ ELISpot reactivity to peptide pools prior to vaccination will be excluded from the analysis of the immunogenicity outcomes.

8.1 Sample size

Among 20 participants assigned to DNAx2 in EV02 30% responded to env plus at least one of gag, pol or nef. Assuming the same proportion in the DNAx2 group (control group) in EV03/ANRS Vac20, then 63 participants per group would provide 90% power to detect a difference between DNAx2 and DNAx3, if in DNAx3 the proportion responding to env plus gag/pol/nef is 60%, an absolute difference of 30%. An absolute difference of 25% (30% vs 55%) would be detected with 80% power.

It is proposed to recruit 70 to each group, 140 in total, to allow for loss to follow-up, and failure to complete the allocated regimen for other reasons.

8.2 Primary analysis

The immunological end-points will be considered present or absent according to standardised operating procedures which take the background activity into account (> 4-fold the negative control and > 55 Spot forming units/10^6 cells). In both EV01 and EV02, a small proportion (<5%) of subjects showed persistent IFNγ ELISpot reactivity to peptide pools prior to vaccination and subsequently across several timepoints. Subjects with IFNγ ELISpot reactivity to peptide pools prior to vaccination will be considered to have non-specific cross-reactive responses to the peptide pools, and will be excluded from the immunogenicity analysis. The two groups DNAx2 and DNAx3 will be compared in terms of the proportion responding to *env* plus *gag/pol/nef* using a Chi-square test.

All safety end-points will be graded by the Clinical Investigators and reviewed by the Operational Sub-Group. Any queries about grade and relationship to study product that cannot be resolved will be referred to the Trial Coordinating Committee for a final decision. For the primary analysis of safety endpoints (as defined in section 1.5.1), results will be expressed as a proportion and the two groups compared using Fisher's exact test.

8.3 Secondary analysis

8.3.1 Immunogenicity

The proportion of participants will be compared at weeks 26 and 28 according to the three categories of response, i.e 1. no response, 2. response to env or 3. response to env+gag/pol/nef or to gag/pol/nef alone as follows. The two groups (DNAx2 and DNAx3) will be compared in terms of the overall response by fitting a multinomial logistic model to the response categories and testing the significance of inclusion of the vaccine assignment in the model. This provides a global test of the null hypothesis that the proportion in the three response categories in DNAx3 is the same as in DNAx2. If this global test was significant the two groups will be compared in terms of the proportion responding to env alone and in terms of the proportion responding to gag/pol/nef (relative to no response).

If present, the response will be quantified in spot-forming units per million cells for each peptide pool at each time-point according to standardised operating procedures. For the purposes of comparison in the secondary analysis, the mean positive response for each regimen will be calculated across the peptide pools and across the following weeks:

- Weeks 26 and 28

The mean percentages CD4 and CD8 T cells responses specific to HIV peptide pools will be evaluated at weeks 26 and 28, 48 and 72 by flow cytometry. HIV specific T cell responses will be defined by the percentages of cells producing IL-2/Interferon-g within CD4 and CD8 T cell populations after ex-vivo stimulation with peptide pools.

8.3.2 Safety

All clinical events and routine laboratory data will be included in the safety analysis. The following tables will be prepared:

- Baseline characteristics, broken down by centre
- The number (percentage) of participants with any solicited local or systemic event commencing in the first 7 days, tabulated by maximum grade and immunisation timepoint
- The number (percentage) of participants with any solicited local event commencing in the first 7 days, tabulated by maximum grade and immunisation timepoint
- The number (percentage) of participants with any solicited systemic event commencing in the first 7 days, tabulated by maximum grade and immunisation timepoint
- The number of episodes (episode of maximum grade, all episodes) of any solicited events commencing in the first 7 days, tabulated by immunisation timepoint
- The number of episodes (episode of maximum grade, all episodes) of solicited local events commencing in the first 7 days, tabulated by immunisation timepoint
- The number of episodes (episode of maximum grade, all episodes) of solicited systemic events commencing in the first 7 days, tabulated by immunisation timepoint
- The number (percentage) of participants with solicited local or systemic events commencing in the first 7 days, tabulated by maximum grade per event per immunisation timepoint
- The number of solicited events per participant during follow-up
- The median (range) duration of solicited events (all combined) by immunisation
- The number (percentage) of participants with any non-solicited event, tabulated by maximum grade and immunisation timepoint
- The number of all non-solicited events including recurrences (number of participants), tabulated by maximum grade and immunisation timepoint
- The number of non-solicited events including recurrences (number of participants), tabulated by maximum grade and immunisation timepoint and limited to those considered possibly, probably or definitely related to study product
- The median (range) duration of non-solicited events (all combined) by immunisation
- The number (percentage) of participants with laboratory events, tabulated by maximum grade per event per immunisation timepoint

Participants who did not receive the immunisation will be excluded from that immunisation timepoint in the solicited event tables. All participants who received an immunisation at any timepoint will be included in the non-solicited event tables. Tables will also be prepared for those who received the full immunisation schedule.

9 Confidentiality, ethics and responsibilities, including indemnity

Full medical confidentiality will be preserved.

The study will be conducted according to ICH GCP guidelines and the Declaration of Helsinki (version 2004) and all applicable local regulations, and it is the responsibility of the Clinical Investigators and the staff at the MRC CTU and INSERM U593 CTU to abide by this protocol.

For EuroVacc centres, the Principal Investigators at each centre are responsible for obtaining the appropriate Local Research Ethics Committee (LREC) approval for the study protocol, the subject information sheet and the consent form. An ethical approval letter stating the title, protocol number and date must be provided to the appropriate data management centre

before study materials will be shipped to the clinical centre. The site Principal Investigators are responsible for informing the LRECs of any SAEs as required and submitting annual reports as required. For ANRS centres, ANRS is responsible for obtaining the approval from the Ethics Committee. ANRS pharmacovigilance team will notify the Ethics Committee of any SAEs as required.

Regulatory submissions will be made in France, Germany, Switzerland, UK and possibly South Africa. The submissions will be coordinated by the EuroVacc Foundation or ANRS, in consultation with Professor Wolf from the University of Regensburg, Sanofi Pasteur and a regulatory consultant where necessary. Regulatory approval must be provided before study materials will be shipped to the clinical centre. Transgene will be responsible for manufacture and labelling of NYVAC-C and Cobra for the DNA-C.

MRC CTU will be responsible for preparing the randomisation list. Each CTU will be responsible for monitoring of the clinical sites and management of the clinical centres affiliated to them (EuroVacc centres for MRC CTU and French centres for INSERM U593 CTU). INSERM U593 CTU will be responsible for data merger of both databases and merger with laboratory data from CHUV for ELISpot; Equipe INSERM for flow cytometry. Both CTUs will have access to this database and will run parallel safety analyses and compare results as part of validation. INSERM U593 CTU, in collaboration with MRC CTU, will be responsible for drafting main report and supplementary reports.
Staff at MRC CTU and ANRS Pharamacovigilance will be responsible for coordinating the response to any SAEs that arise during the course of the trial and reporting these if indicated to the regulatory authorities, and to the Sponsors in the appropriate time-frames.

The EuroVacc Foundation will sponsor the trial in the EuroVacc centres. The ANRS will sponsor the trial in the ANRS centres. The EuroVacc Foundation and the ANRS will coordinate the necessary clinical trial agreements delineating the above responsibilities and the liability for events occurring as a result of participating in the trial.

Responsibility for indemnity will be ensured by the following parties:

- In the EuroVacc centres, the EuroVacc Foundation will provide insurance for harm due to trial participation through a clinical trial liability. This will not provide cover for harm due to clinical negligence or negligence during the manufacture of product.
- In the ANRS centres, the ANRS will provide insurance for harm due to trial participation through a clinical trial liability. This will not provide cover for harm due to negligence during the manufacture of product.
- Each clinical centre will be responsible for ensuring that cover is in place for harm due toclinical negligence caused by their employees to a trial participant

10 Publication

It is intended that the results of this study will be published in an appropriate peer-reviewed journal, with the aim of submitting a paper for publication within 12 months of the study's completion. The Trial Coordinating Committee will have 30 days to comment on any manuscript. No other publications, wither in writing or verbally, will be made before the definitive manuscript has been agreed and accepted for publication, without the prior approval of this committee.

A final report of the study will be prepared by the Investigators and circulated to all parties involved in the trial for comments.

Trial data will be made available to Sanofi Pasteur, the EuroVac consortium and Cobra in an agreed format at the end of the trial.

11 References:

1 Myers G. Los Alamos National Laboratory
2 Korber B. et al. AIDS 1998; 12: 256
3 Weber J. et al. J Virology 1996; 70: 7827

4 Moore JP. Et al. J Virology 1996; 70: 427
5 Nyambi P. et al. J Virology 1996; 70: 6235
6 Gotch F. et al. AIDS 1998; 12: 447
7 Koup R. et al. J Virology 1998; 72: 4016
8 Excler J-L and Plotkin S. AIDS 1997; 11: S127
9 McMichael AJ, Hanke T. Nat Med 1999; 5:612-614.
10 McMichael AJ, Rowland-Jones SL. Nature 2001; 410:980-987
11 Baba T. et al., Science 1995; 167: 1822
12 Stott J. et al., Nature 1991;
13 Beddows S. et al., J Virology 1999; 73: 1740
14 The rgp120 HIV Vaccine Study Group. JID 191(5):654-65
15 Redfield RR et al. NEJM 1987; 316: 673
16 Ockenhouse CF et al. JID 1998; 177: 1664
17 Keefer MC et al. Int Conf AIDS 1998; 12: 278
18 Graham BS et al. JID 1992; 166:244
19 Cranenburgh R et al. Nucleic Acid Res 2001 29(5):E26
20 Su L, Graf M, von Briesen H, Xing H, Kostler J, Melzl H, Wolf H et al. J Virol 2000 74:11367-76
21 Deml L, Bojak A, Steck S, Graf M, Wild J, Schirmbeck R et al. J Virol 2001 75:10991-11001
22 Graf M, Bojak A, Deml L, Wolf H, Wagner R. J Virol 2000 74:10822-6
23 Wagner R, Graf M, Bieler K, Wolf H et al. Human Gene Therapy 2000 11:2403-13
24 Mooij P et al., J Virology 2004 78: 3333-3342
25 Kanesa-thasan N et al. Vaccine 2000 19(4-5):483-91
26 RA Koup BS Graham, M Roderer, R Bailer et al Antiviral Therapy 2006 11:supplement 2 S07-03
27 Rodger MA, King L. J Advanced Nursing 2000 31(3): 574-582
28 Advisory Committee on Immunization Practices (ACIP) General recommendations on immunization MMWR 1994;43:RR-1:6

Appendix 1 Information Sheet for all Participants
Hospital headed note paper
EuroVacc 03 (EV03) / ANRS Vac20
A trial to compare the immune responses and safety of two versus three DNA HIV vaccine primes in combination regimens with a pox vector boost in healthy volunteers

This document could be adapted to comply with local rules and regulations

General notes about participating in medical research
You are being invited to take part in a research study. Before you decide it is important for you to understand why the research is being done and what it will involve. Please take time to read the following information. You are encouraged to talk it over with your partner, and it may also be helpful to talk to close friends, and your family doctor but you are not obliged to do this. Please ask us if there is anything that is not clear or if you would like more information. Take time to decide whether or not you wish to take part.

Thank-you for reading this. Eurovacc03/ANRS Vac 20 (2.0) 25th October 2006
No changes required to support protocol 2.2 of 16th January 2007

What is the purpose of the study?
There is an urgent need to develop methods to prevent HIV infection, given the continuing world-wide epidemic and an estimated 14,000 new infections a day. To date, several HIV vaccines have been developed, and two have been assessed in a large population at risk of HIV infection. Unfortunately the results of these studies, the first of their kind, were disappointing as the vaccines were not effective. Research teams have continued to develop new products and approaches, some of which look more promising in the laboratory.

This is the second trial of DNA HIV-C prime using human volunteers, and the third assessment of NYVAC HIV-C. Although safety is still an important purpose of the study, it will also be possible to compare the immune responses by examining blood taken from volunteers in each of two groups. This will help to determine whether three DNA HIV-C primes lead to broader immune responses than two primes.

Who is organising and funding the research?
The research is sponsored by the EuroVacc Foundation and the ANRS with funding from a variety of sources including the European Commission and ANRS. The study will be coordinated by the UK Medical Research Council (MRC) Clinical Trials Unit in London, and the INSERM U593 Clinical Trials Unit in Bordeaux, France.
It will be conducted according to Good Clinical Practice guidelines based on internationally accepted standards, in centres throughout Europe (France, Germany, Switzerland and UK), and possibly South Africa.

Who has reviewed the study?
The study has been reviewed by the Local Research Ethics Committee appropriate to your clinical centre and by the authority that supervises the use of medicines and gene therapy in the countries involved.

What are the products being tested?
There are 2 different vaccine products.

(i) DNA HIV-C
(ii) NYVAC HIV-C

140 individuals will receive both products, half will receive 3 DNA HIV-C + 1 NYVAC HIV-C and half will receive 2 DNA HIV-C + 2 NYVAC HIV-C.

The NYVAC HIV-C is based on the vaccinia (pox) virus, which was used as a vaccine in the prevention of smallpox. The part like vaccinia is called NYVAC and this acts as a carrier for the genetic code for certain HIV proteins that we hope will produce a broad immunity to HIV. There are several different types (strains, clades) of HIV circulating in the world. The most usual one in Europe is clade B. Clade C is more common in sub-Saharan Africa, India and China. A clade C virus has been used to design the genetic code in NYVAC HIV-C. The NYVAC has been made much safer than vaccinia virus by taking out certain genes that are critical for it to replicate or cause harm. NYVAC has been tested in a small number of humans in immunisation trials carrying other products, and judged to be safe. 20 volunteers were immunised with NYVAC HIV-C, in the EV01 study. The volunteers received 2 injections 4 weeks apart and no severe reaction to the product was observed. The most common side-effects observed were similar to those seen with licensed vaccines, e.g.: pain and redness at the injection site, headache and malaise. These effects were mild or moderate and in the majority lasted one to three days. A further 35 volunteers have received 2 injections of NYVAC HIV-C in the EV02 study and there have been 2 severe local reactions (1 swelling, 1 pain) both of which resolved within 7 days.

The DNA HIV-C consists of two plasmids (pieces of DNA), together representing the genetic code that is inserted into the NYVAC described above. There have been many other studies of DNA products in man now, which have shown that DNA is well-tolerated and safe. However, prior to EV02, none had used two plasmids and none had been based on clade C HIV virus. Recently reported work suggests that higher doses of DNA are more likely to produce an immune response. Because it is not technically possible to concentrate the DNA HIV-C, we need to give a larger volume than usual, and so each DNA HIV C immunisation will be two injections, one in each thigh. In EV02 volunteers received two immunisations, 4 weeks apart and in this study we have added a third for half of the participants. We would like to see whether the third immunisation increases the proportion of volunteers that develop immune responses.

What are the side-effects?

There may be pain, redness and swelling at the place you are injected or you may develop a rash such as small blisters. Also, you could have fever, chills, a more widespread rash, aches and pains, nausea, headache and excess fatigue. We know that these side-effects can happen with other vaccines, so they may occur with the ones in this study. However they usually don't last long and don’t require treatment to be prescribed. There could be other side-effects that we don't know about yet, but this is unlikely.

Your blood may register a positive result in a routine HIV test, because of your immune response to the vaccine. However, the routine laboratory attached to the centre you are attending will be able to distinguish between real infection and a post-immunisation response by using more than one test. At the end of the study the staff will tell you the result of the routine HIV test and if your blood is still registering positive in any of the tests, you will be

given a card and access to ongoing HIV testing through the clinic. You will be recalled on a yearly basis until your blood no longer registers positive in any of the routine tests. You should refrain from donating blood until the routine screen for HIV has become negative.

Why have I been asked to participate?
The researchers are looking for 140 people to take part in various centres throughout France, Germany, Switzerland and the United Kingdom and possibly South Africa.

You can participate because you are:

- ☺ a healthy male or female aged between 18 and 55 years
- ☺ available for 13 visits over 48 weeks and then one later visit at 18 months
- ☺ at low risk of HIV, willing to remain so for the study, and willing to undergo a HIV test
- ☺ (if relevant) using an effective method of contraception with your partner, willing to continue with this during and for 4 months after the immunisation period, and willing to undergo pregnancy tests
- ☺ willing to receive 4 immunisations (6 or 7 injections)
- ☺ willing to undergo a genital infection screen
- ☺ able to give written informed consent

You could not take part if you:

- ☹ are pregnant or breast-feeding
- ☹ have an autoimmune disease, immunodeficiency illness or have taken immunosuppressive drugs in the last 3 months, for example steroids
- ☹ have grand-mal epilepsy, or are taking anti-epileptics
- ☹ have severe allergic disease, severe eczema or allergy to eggs or to gentamicin
- ☹ had a severe local or general reaction to vaccination in the past
- ☹ have abnormalities in your routine blood tests collected at screening
- ☹ are found to be HIV positive at screening
- ☹ are taking part in another clinical trial of an unlicensed product
- ☹ have had a 'live' vaccine within the last 60 days or blood products within 4 months
- ☹ have active B or C hepatitis or uncontrolled infection
- ☹ have haematological, metabolic, renal, gastro-intestinal or liver disorders
- ☹ are unlikely to comply with protocol

Do I have to take part?
It is up to you to decide whether or not to take part. If you do decide to take part you will be asked to sign a consent form. You are still free to stop at any time. This will not affect the way you are treated at this hospital, should you attend in the future.

What will happen to me if I take part?
Firstly you will attend for a screening visit. This is an opportunity for you to have all your questions answered. The doctor or nurse for the study will check that you are eligible to join the study. This will involve general health questions as well as some more personal questions, an examination and some blood tests, including a HIV test. You will be asked to go away and think carefully about the study. If you decide to join you will be asked to attend hospital on at least 12 further occasions over 48 weeks, then at 18 months.

You will receive four immunisations, the first two into the muscles of your legs (2 injections each time), and then either one more (2 injections) in your legs and one into the muscle of your arm or two into the muscle of your arm.

Everyone will be asked to record their symptoms for at least 7 days after each immunisation. Other visits will involve answering questions about your health, examination of the injection site, and blood tests. Less than 1000ml of blood (equivalent to two donations) will be drawn over the period of the study. Most of this blood will be tested straight away, but some will be stored for future analysis. Tissue typing will also be done on your blood to assist the immune tests.

Site specific statements to be inserted stipulating details of any reimbursements to be given to participants for travel or time.

What do I have to do?

- You need to continue to stay at low risk of catching HIV infection.
- If you need a HIV test during the study, you should ask the study staff who will organise for this to be done.
- As we are at a very early stage in the investigation of these products, you (or your partner) should not become pregnant until 4 months after your last immunisation.
- It is important that you try to attend according to the schedule, especially the visits just after an immunisation.
- As this product is new, we would like to monitor your health over a longer period. We can do this by contacting you directly each year. However people often move and it would be ideal if we could follow you up through your GP or by cross-checking your name against the residential registry. You can participate in the trial without agreeing to do this and can withdraw your consent at any time.

What if something goes wrong?

If you suffer physical, psychological or social harm as a result of participating in this trial, you should speak to one of the trial team. There are 3 parts to the compensation in place: cover for harm due to a manufacturing fault, cover for harm caused by staff, and cover for harm due to participation but which is not due to either the manufacture of products or the staff. The trial staff will advise you on how to proceed with a complaint or claim. Any decisions about your case will be made by independent authorities.

If you have a complaint about the trial staff then you should direct this to the Principal Investigator or Hospital Authority.

In the event of harm which occurs after the trial has finished, you should contact the clinical centre. If the centre appears to have closed or relocated, then contact the Hospital or University Authorities. In the unlikely event that these have both closed, you should contact either the UK Medical Research Council (+44 (0) 20 7636 5422) or the ANRS as these government agencies employ staff responsible for the coordination of the trial.

What is the current situation with HIV vaccine research?

At the moment there are several new HIV vaccines being developed throughout the world, the majority at an early stage of assessment. Two vaccines have recently been tested in large populations, one in the US and one in Thailand, to see if they protect against HIV infection. Both of these were simple synthetic proteins, which is the class researchers have the most experience with. They were well-tolerated, but did not lead to very strong immune responses. Overall, there was no protection in the vaccinated individuals in either trial.

What if new information becomes available?
If the new information is relevant to this trial, the study team will tell you about it.

Will my taking part be confidential?
Yes. Only the study staff will know your name, and it will only appear in documents held at the clinical centre. Information held at the data management centres (MRC Clinical Trials Unit and INSERM) will be identified only by a trial number, a hospital number and date of birth. Participants will be identified in the analysis files by trial number and date of birth, including those reviewed by the independent monitoring committees. There will be no identifiers in scientific publications or presentations.

What will happen to the results?
After the study is completed there will be an analysis. This usually takes 3-6 months and after this you will be told the results of the study. The results of the study will be written up and submitted for review by a medical journal. They may also be presented at scientific conferences.

What are the possible benefits?

- you will have a general check-up as part of the screening and the opportunity to discuss your health
- you will also receive information about sexually transmitted infections including HIV and how to prevent them
- the information that we get from this study may help us to develop an effective HIV vaccine which would benefit a large proportion of the world's population

What are the possible disadvantages and risks of taking part?

- there is a possibility of physical side-effects, and that the vaccine could harm an unborn child if given close to the time of conception or in the early part of pregnancy
- if your blood does react positive in the standard HIV test, there is a possibility of social discrimination. This has not been reported in other HIV vaccine studies.
- there is a chance that you will mistakenly believe that you are protected against HIV and put yourself at risk as a consequence. It is important to remember that NO-ONE knows yet whether these products will prevent HIV
- there is a theoretical chance that receiving this vaccine could make HIV infection progress more rapidly if you were to catch it in the future, but this has not happened in the HIV vaccine trials conducted so far
- taking this test vaccine might mean that you will not be able to take part in any other trials of potential AIDS vaccines, although you would not be prevented from having an effective HIV vaccine if such a vaccine became available commercially. However, if such a vaccine were based on NYVAC, it might be less effective in you. This could only happen if your immune system was still able to remember the NYVAC in this trial.

For further information, please contact:

Trial Physician ______________________________

Trial Nurse ______________________________

Thank-you for taking the time to read this

You should keep a copy of this information sheet, and your signed informed consent if you decide to join the study.

Appendix 2 Informed Consent

This document could be adapted to comply with local rules and regulations

Hospital headed note paper

EuroVacc 03/ANRS Vac 20 Trial Informed Consent
Protocol version (2.2)

Screening Number		Date of birth.	

The subject must complete the following questions themself

Part 1 of Informed Consent – consent for screening

PLEASE CIRCLE THE CORRECT ANSWER

Has the EuroVacc 03/ANRS Vacc 20 trial been explained to you and have you been given a Participant Information sheet dated 25th October 2006? YES / NO

Have you had an opportunity to ask questions and discuss this trial? YES / NO

Have you received satisfactory answers to all of your questions? YES / NO

Are you aware that in order to be accepted in this study you will have to have an HIV test before and during the study? YES / NO

Are you aware that participants must use effective contraception if relevant, during and for 4 months after the immunisations? YES / NO

Do you agree to appropriate members of trial staff and the authorities responsible for licensing the vaccines having access to your medical records? YES / NO

Which study doctor or nurse have you spoken to about this trial?

....

PLEASE PRINT HIS/HER NAME

Do you understand that you are free to withdraw from the trial:

- at any time without having to give a reason

- and without affecting future medical care? YES / NO

Do you agree to be screened for this trial? YES / NO

SIGNATURE OF VOLUNTEER AND DETAILS

Signature		Date of signature	
Print name			

SIGNATURE OF INVESTIGATOR AND DETAILS:

Signature		Date of signature	
Print name			

Please store one copy of this form in the clinical records

Hospital headed notepaper
EuroVacc 03/ANRS Vac 20 Trial Informed Consent
Protocol version (2.2)

Screening Number [ ] Date of birth. [ ]

The subject must complete the following questions themself

Part 2 of the Informed Consent – consent for enrolment

PLEASE CIRCLE THE CORRECT ANSWER

Have you read the EuroVacc 03/ANRS Vac 20 Participant Information sheet dated 25th October 2006? YES / NO

Have you received enough information about the trial? YES / NO

Do you understand that it is not known whether the vaccines to be tested can prevent HIV infection? YES / NO

Do you understand that coded information about you will be held electronically and available to additional investigators working on the trial? YES / NO

Do you agree that stored specimens from this trial can be tested in future for immunity to HIV and the NYVAC? YES / NO

Do you agree to take part in this trial? YES / NO

Are you happy to be contacted annually for long-term follow-up? YES / NO

Do you agree to investigators from the clinical centre accessing the residential registry in your country in order to assist in long-term follow-up? YES / NO

SIGNATURE OF VOLUNTEER AND DETAILS

Signature		Date of signature	
Print name			

SIGNATURE OF INVESTIGATOR AND DETAILS:

Signature		Date of signature	
Print name			

Please store one copy of this form in the clinical records

Appendix 3 Packaging and labelling

To be added when finalised

Appendix 4 Grading of clinical and laboratory adverse events

Based on systems in use at the MRC CTU, IAVI and NIH Division of AIDS

Abbreviations:				
	ULN	Upper Limit of Normal	LLN	Lower Limit of Normal
	R_x	Therapy	Req	Required
	Mod	Moderate	IV	Intravenous
	ADL	Activities of Daily Living	Dec	Decreased

For events not specified in the tables below the following grading should be applied:

Grade 1 (mild)	No medical intervention required and/or minimal effect on daily activities such that slightly reduced for no more than 48hrs
Grade 2 (moderate)	Repeated medication or medical intervention required and/or activity reduced up to half usual level for more than 48 hrs
Grade 3 (severe)	Extensive/prolonged medical intervention or repeated prescribed medication; bed rest or activity reduced by >50% of usual level; can't work
Grade 4 (extreme)	Hospitalisation and/or life-threatening

LABORATORY PARAMETERS

PARAMETER	GRADE 1<br>MILD	GRADE 2<br>MODERATE	GRADE 3<br>SEVERE	GRADE 4<br>EXTREME
**HAEMATOLOGY**				
Hb	10.0-10.9 g/dL	9.0-9.9 g/dL	7.0-8.9 g/dL	<7.0 g/dL
White Blood Count	13.0 – 14.9 x10^9/l<br>or 2 – 2.5 x10^9/l	15.0 – 19.9 x10^9/l<br>or 1.5 – <2.0 x10^9/l	20.0 – 29.9 x10^9/l<br>or 1 – <1.5 x10^9/l	≥30.0<br>or <1.0 x10^9/l
Absolute Neutrophils	1.3-1.0 x10^9/l	<1.0-≥0.75 x10^9/l	<0.75-≥0.5 x10^9/l	<0.5 x10^9/l
Percent neutrophils	>80%	90%	≥95%	---
Lymphocytes	0.7- 0.899 x10^9/l	0.5-0.699 x10^9/l	0.35-0.499 x10^9/l	<0.35 x10^9/l
Platelets	100 –124.999 x 10^9/l	50 – 99.999 x 10^9/l	25 – 49.999 x10^9/l	<25.0 x10^9/l
CD4 Count	300-400/mm^3	<300mm^3	<200/mm^3	<100/mm^3
**BIOCHEMISTRY**				
Potassium<br>Hyperkalemia<br>Hypokalemia	 5.6 – 6.0 meq/L<br>3.0 – 3.4 meq/L	 6.1-6.5 meq/L<br>2.5 – 2.9 meq/L	 6.6-7.0 meq/L<br>2.0– 2.4 meq/L	 >7.0 meq/L<br><2.0 meq/L
Bilirubin<br>Hyperbilirubinemia	 >1.25 – 2.0 x ULN	 >2.0 – 2.5 x ULN	 >2.5 – 5 x ULN	 >5 x ULN
Glucose<br>Hypoglycaemia<br>Hyperglycaemia nonfasting; no prior diabetes	 2.3-2.4 mmol/l<br>7.0-10.0 mmol/l	 2.1-2.2 mmol/l<br>10.1-15.0 mmol/l	 1.5-2.0 mmol/l<br>15.1-25.0 mmol/l	 <1.5 mmol/l<br>>25.0 mmol/l
Transaminases<br>AST (SGOT)<br>ALT (SGPT)<br>GGT<br>Alk Phos	 1.25 – 2.5 x ULN<br>1.25 – 2.5 x ULN<br>1.25 – 2.5 x ULN<br>1.25 – 2.5 x ULN	 >2.5 – 5.0 x ULN<br>>2.5 – 5.0 x ULN<br>>2.5 – 5.0 x ULN<br>>2.5 – 5.0 x ULN	 >5.0 – 10.0 x ULN<br>>5.0 – 10.0 x ULN<br>>5.0 – 10.0 x ULN<br>>5.0 – 10.0 x ULN	 > 10.0 x ULN<br>> 10.0 x ULN<br>> 10.0 x ULN<br>> 10.0 x ULN
Amylase	>1.0 – 1.5 x ULN	>1.5 – 2.0 x ULN	>2.0 – 5.0 x ULN	>5.0 x ULN
Creatinine	130-180µmol/l	181-360µmol/l	361-720µmol/l	>720µmol/l
**URINALYSIS**				
Proteinuria:<br>24 hour urine	200 mg - 1 g loss/day OR <0.3% OR <3 g/l	1 – 2 g loss/day OR 0.3 – 1.0% OR 3 - 10 g/l	2 – 3.5 g loss/day OR >1.0% OR > 10 g/l	Nephrotic syndrome OR >3.5 g loss/day
Haematuria	Microscopic only ≤10 RBC/HPF	>10 RBC/HPF	Gross, with or without clots OR RBC casts	Obstructive OR transfusion req

CLINICAL PARAMETERS

PARAMETER	GRADE 1 MILD	GRADE 2 MODERATE	GRADE 3 SEVERE	GRADE 4 EXTREME
**CARDIOVASCULAR**				
Cardiac Arrhythmia		Asymptomatic; transient dysrhythmia, no R_x req	Recurrent/persistent dysrhythmia; symptomatic R_x req	Unstable dysrhythmia, hospitalisation and R_x req
Hypertension	Transient, increase >20 mm Hg diastolic BP; no $R_x$req	Recurrent; chronic increase >20 mm Hg diastolic BP; R_x req	Acute R_x req; outpatient	Hospitalisation req OR end organ damage
Hypotension	Transient orthostatic hypotension with heart rate increased by >20 beats/min OR decreased by > 10 mm Hg systolic BP, no R_x req	Symptoms OR BP decreased by >20 mm Hg systolic, correctable with oral fluid R_x	IV fluid req	Mean arterial pressure <60 mm Hg, OR end organ damage, OR shock, vasopressor R_x req OR hospitalisation
Pericarditis	Minimal effusion	Mild/mod asymptomatic effusion, no R_x	Symptomatic effusion, pain, EKG changes	Tamponade OR pericardiocentesis OR surgery req
Haemorrhage, blood loss		Mildly symptomatic, no R_x req	Gross blood loss OR 1-2 units transfused	Massive blood loss OR >2 units transfused
**GASTROINTESTINAL**				
Diarrhoea	Mild OR transient; 3-4 loose stools per day OR mild diarrhoea lasting <1 week	Mod OR persistent; 5-10 loose stools per day OR diarrhoea lasting $\geq$1 week	>10 loose stools/day bloody diarrhoea; OR orthostatic hypotension OR electrolyte imbalance, >2 L IV fluid req	Hypotensive shock OR severe electrolyte imbalance
Oral Discomfort/ Dysphagia	Mild discomfort, no difficulty swallowing	Difficulty swallowing but able to eat and drink	Unable to swallow solids	Unable to drink fluids; IV fluids req
Constipation	------------	Moderate abdominal pain 78 hours with impaction require outpatient prescription	Requiring disimpaction or hospital treatment	Distention with vomiting OR obstipation
**PULMONARY**				
Cough (for aerosol studies)	Transient; no R_x	Treatment associated cough; inhaled bronchodilator	Uncontrolled cough; systemic R_x req	---------------
Bronchospasm Acute	Transient; no R_x; FEV1 or peak flow reduced to 70% - 80%	R_x req; normalizes with bronchodilator; FEV1 or peak flow 50% - 69%	No normalization with bronchodilator; FEV1 or peak flow 25% - 49%, retractions	Cyanosis; FEV1 or peak flow <25% OR intubated
Dyspnoea	Dyspnoea on exertion	Dyspnoea with normal activity	Dyspnoea at rest	Dyspnoea requiring O_2 therapy
**NEUROLOGICAL**				
Neuro-cerebellar	Slight incoordination OR Dysdiadochokinesia	Intention tremor OR dysmetria OR slurred speech OR nystagmus	Ataxia requiring assistance to walk or arm incoordination interfering with ADLs	Unable to stand
Neuro-psych/mood	---------------	---------------	Severe mood changes requiring medical intervention; suicidal ideation	Acute psychosis req hospitalisation ; suicidal gesture/attempt
Parasthesia (burning, tingling, etc.)	Mild discomfort; no R_x req	Mod discomfort; non-narcotic analgesia required	Severe discomfort; OR narcotic analgesia req with symptomatic improvement	Incapacitating; OR not responsive to narcotic analgesia

PARAMETER	GRADE 1 MILD	GRADE 2 MODERATE	GRADE 3 SEVERE	GRADE 4 EXTREME
Neuro-motor	Mild weakness in muscle of feet but able to walk and/or mild increase or decrease in reflexes	Mod weakness in feet (unable to walk on heels and/or toes), mild weakness in hands, still able to do most hand tasks and/or loss of previously present reflex or development of hyperreflexia and/or unable to do deep knee bends due to weakness	Marked distal weakness (unable to dorsiflex toes or foot drop, and mod proximal weakness e.g., in hands interfering with ADLs and/or requiring assistance to walk and/or unable to rise from chair unassisted	Confined to bed or wheel chair because of muscle weakness
Neuro-sensory	Mild impairment (decreased sensation, e.g., vibratory, pinprick, hot/cold in great toes) in focal area or symmetrical distribution	Mod impairment (mod decreased sensation, e.g., vibratory, pinprick, hot/cold to ankles) and/or joint position or mild impairment that is not symmetrical	Severe impairment (decreased or loss of sensation to knees or wrists) or loss of sensation of at least mod degree in multiple different body sites (i.e., upper + lower extremities)	Sensory loss involves limbs and trunk
Eye		Mild pain, visual changes, conjunctivae erythema, abnormal slit lamp	Loss of vision, clinically diagnosed uveitis, mod-severe pain, glaucoma	---------------
**MUSCULOSKELETAL**				
Arthralgia/Arthritis	Arthralgia	Arthralgia with joint effusion or moderate impairment of activity	Frank arthritis with or without effusion OR resulting in severe impairment of activity	Hospitalisation
Myalgia	Myalgia without limitation of activity	Muscle tenderness at other than injection site or with moderate impairment of activity eg difficulty climbing stairs	Frank myonecrosis OR with severe impairment of activity eg can't climb stairs	Hospitalisation
**OTHER SOLICITED VACCINE REACTIONS**				
Pain at injection site	Mild, no Rx required or analgesics for mild-moderate pain	Moderate requiring regular analgesics for mild-moderate pain or occasional analgesics for moderate-severe pain	Severe, repeated analgesics for moderate-sever pain	Hospitalisation
Fever Oral>12 hours	37.7 - 38.9°C (100.0 – 101.5°F)	39.0 – 39.7°C (101.6 – 102.9°F) OR max temp of 103°F	39.8 – 40.5°C (103 - 105°F) OR max temp of 103.5°F	>40.5°C (105°F) OR max temp of >105°F
Headache	Mild, no R_x req, OR or analgesics for mild-moderate pain	Mod requiring regular analgesics for mild-moderate pain or occasional analgesics for moderate-severe pain	Severe; intractable; OR requiring repeated analgesics for moderate-severe pain	Requiring hospitalisation, or associated with neurological, respiratory or cardiovascular abnormalities
Malaise	Transient,easily tolerated and/or normal activity reduced	Fatigue such that spends ½ day in bed ≤ 2d	Fatigue such that in bed all day or in bed ½ day >2d	Hospitalisation
Chills / rigors	Requiring symptomatic treatment	Prolonged >6hrs requiring regular analgesics for mild-moderate pain or Occasional analgesics for moderate-severe pain	Repeated analgesics for moderate-severe pain	Rigors

CUTANEOUS VACCINE REACTIONS

**Reaction**	**Mild**	**Moderate**	**Severe**	**Extreme**
Immediate reactions (within 6 hours of injection)	Symptoms of irritation locally (usually itching at the injection site)<br>OR<br>Erythema +/- swelling at the injection site		Laryngeal oedema insufficient to require intubation; diarrhoea insufficient to require IV fluids, or asthma insufficient to require hospitalisation<br>OR<br>Urticaria, angio-oedema<br>OR<br>Generalised pruritus	Anaphylactic shock
Delayed reactions	**Erythema at injection site**<br>Erythema up to and including 50% of baseline arm circumference<br>OR<br>Symptoms of irritation that are easily tolerated and do not require repeated medication<br>OR<br>Both	**Erythema at injection site**<br>Erythema greater than 50% of the arm circumference at baseline<br>With or without<br>Symptoms of irritation that do not require repeated medication<br>OR<br>Symptoms of irritation that require repeated medication AND erythema up to and including 50%	**Erythema at injection site**<br>Erythema greater than 50% of the arm circumference at baseline AND symptoms of irritation requiring repeated medication	Hospitalisation
	**Blistering or ulceration at injection site**<br>Fluid filled vesicles or superficial disruption of epithelium covering an area < 1cm	**Blistering or ulceration at injection site**<br>Fluid filled vesicles or superficial disruption of epithelium, area 1 - 2cm<br>OR<br>Blood filled vesicles<br>OR<br>Full thickness disruption of epithelium healed within 2 weeks	**Blistering or ulceration at injection site**<br>Full thickness disruption of epithelium not healed within 2 weeks	**Necrosis**
Intramuscular vaccination	**Soft swelling – local**<br>Swelling <25% of arm or leg	**Soft swelling – local**<br>Swelling 25-50%of arm or leg	**Soft swelling – local**<br>Swelling >50% of arm or leg<br>**Or**<br>**Induration/hardened swelling**<br>**(when considered by the clinician to be associated with a process arising in the muscle)**	
Intradermal vaccination, or attributable to leakage of material into the dermis after intramuscular vaccination	**Induration/hardened swelling**<br>Induration<br>Diameter < 1.5cm	**Induration/hardened swelling**<br>Induration<br>Diameter 1.5 – 3cm<br>And<br>Lasting for > 6 days	**Induration/hardened swelling**<br>Induration<br>Diameter > 3cm<br>And<br>Lasting for > 6 days	**Necrosis**
	**Generalised rash**<br>***Scattered (< 5 sites) maculo-papular rash AND no symptoms***	**Generalised rash**<br>Scattered macular or papular rash AND symptoms<br>OR<br>*Widespread rash AND not requiring repeated medication for symptoms*	**Generalised rash**<br>*Widespread rash AND symptoms requiring repeated medication*<br>OR<br>Involving mucous membranes (Stevens-Johnson reaction)	**Generalised rash**<br>Exfoliative dermatitis

Appendix 5 Diary Card
Guide for grade of general reaction

	Grade 1<br>mild	Grade 2<br>moderate	Grade 3<br>severe	Grade 4<br>Extreme
Chills/rigors	*Mild hot/cold flush requires blanket or occasional aspirin/paracetamol*	*Limiting daily activity >6 hours, or need regular aspirin/paracetamol*	*Uncontrollable shaking, treatment from doctor needed*	*Hospitalisation*
Malaise/abnormal tiredness	*Normal activity reduced – not bad enough to go to bed*	*Fatigue such that ½ day in bed for 1 or 2 days*	*Fatigue such that in bed all day or ½ day for more than 2 days*	*Hospitalisation*
General (all over) muscle aches	*No limitation of activity*	*Muscle tenderness, limited activity e.g. difficulty climbing stairs*	*Severe limitation e.g. can't climb stairs*	*Hospitalisation*
Headache	*No treatment or responds to paracetamol like treatment*	*Regular paracetamol like treatment needed*	*Regular strong painkillers treatment needed*	*Hospitalisation*
Nausea	*Intake maintained*	*Intake reduced less than 3 days*	*Minimal intake 3 days or more*	*Hospitalisation*
Vomiting	*Less than 4 x a day or lasting less than 1 week*	*At least 4 x day or lasting 1 week or more*	*Unable to keep any food or fluids down*	*Hospitalisation*

- **Complete at approximately the same time every day ONLY on days that you are NOT going to clinic**
- **Fill in temperature and record the grade of other general reactions (use the table above as a guide)**
- **Add any comments regarding other symptoms**
- **Record any medication taken including over the counter medicines and anything taken to relieve local reactions**
- **Record the grade, or size in cm, of local reactions overleaf**
- **Please contact a member of the trials staff as soon as possible if you experience any symptoms of grade 3 or 4**
- **When the diary card is complete, please return it to a member of trials staff at your next visit**

	**12 hrs**	**Day 1**	**Day 2**	**Day 3**	**Day 4**	**Day 5**	**Day 6**	**Day 7**
General Symptoms								
**Date**								
**Temperature ºC**								
**Chills/rigors**								
**Malaise/ tiredness**								
**General muscle aches**								
**Headache**								
**Nausea**								
**Vomiting**								

COMMENTS

____

____

____

____

____

Medication – list all medications and total daily dose

	**Dose**	**Dose**	**Dose**	**Dose**	**Dose**	**Dose**	**Dose**	**Dose**
**1**								
**2**								
**3**								

Guide for grade of local reactions

	Grade 1 mild	*Grade 2 moderate*	*Grade 3 severe*	*Grade 4 Extreme*
Pain in injected muscle (including ache) NOT discomfort in skin	*No treatment or responds to paracetamol like treatment*	*Regular paracetamol like treatment needed*	*Regular strong painkillers needed*	*Hospitalisation*
Itching or irritation in the skin at the site of injection	*No treatment or responds to paracetamol like treatment*	*Regular paracetamol like treatment needed*	*Regular strong painkillers needed*	*Hospitalisation*

Local Symptoms – please put in grade, including 0 if no symptoms and measure maximum width of skin lesion in cm

	**12 hrs**		**Day 1**		**Day 2**		**Day 3**		**Day 4**		**Day 5**		**Day 6**		**Day 7**	
	left	right	left	right	left	right	left	right	left	right	left	right	left	right	left	right
**Pain at injection site**																
**Itching or irritation at the site**																
**Redness/discolouration (cm)**																
**Fluid filled blisters (cm)**																
**Blood filled blisters (cm)**																
**Hard swelling in skin surface at or close to site (cm)**																

Diary

EUROVACC EV03
ANRS Vacc 20

**Date of birth**	
**Trial number**	
**Trial centre**	
**Vaccination number**	**1 2 3 4**<br>Please circle as appropriate
**Date of vaccination**	

**Contact details:**
